# Spiking Neural Network Models of Interaural Time Difference Extraction via a Massively Collaborative Process

**DOI:** 10.1523/ENEURO.0383-24.2025

**Published:** 2025-07-24

**Authors:** Marcus Ghosh, Karim G. Habashy, Francesco De Santis, Tomas Fiers, Dilay Fidan Erçelik, Balázs Mészáros, Zachary Friedenberger, Gabriel Béna, Mingxuan Hong, Umar Abubacar, Rory T. Byrne, Juan Luis Riquelme, Yuhan Helena Liu, Ido Aizenbud, Brendan A. Bicknell, Volker Bormuth, Alberto Antonietti, Dan F. M. Goodman

**Affiliations:** ^1^Laboratoire Jean Perrin, Institut de Biologie Paris-Seine, CNRS, Sorbonne Université, Paris 75005, France; ^2^Department of Electrical and Electronic Engineering, Imperial College London, London SW7 2AZ, United Kingdom; ^3^School of Psychological Science, University of Bristol, Bristol BS8 1QU, United Kingdom; ^4^Department of Electronics, Information and Bioengineering, Politecnico di Milano, Milano 20133, Italy; ^5^Department of Data Analysis, Ghent University, Ghent 9000, Belgium; ^6^Faculty of Brain Sciences, University College London, London WC1N 3AR, United Kingdom; ^7^School of Engineering and Informatics, University of Sussex, Brighton BN1 9QT, United Kingdom; ^8^Centre for Neural Dynamics and Artificial Intelligence, University of Ottawa, Ottawa, Ontario K1N 6N5, Canada; ^9^Department of Physics, University of Ottawa, Ottawa, Ontario K1N 6N5, Canada; ^10^Department of Computer Science and Engineering, The Chinese University of Hong Kong, Shatin, N.T., Hong Kong SAR, China; ^11^COMBYNE lab, University of Surrey, Guildford GU2 7XH, United Kingdom; ^12^Department of Engineering, University of Cambridge, Cambridge CB2 1PZ, United Kingdom; ^13^Max Planck Institute for Brain Research, Frankfurt 60438, Germany; ^14^School of Life Sciences, Technical University of Munich, Freising 85354, Germany; ^15^Princeton Neuroscience Institute, Princeton University, Princeton 08540, New Jersey; ^16^Department of Applied Mathematics, University of Washington, Seattle WA 98105, Washington; ^17^Edmond and Lily Safra Center for Brain Sciences (ELSC), The Hebrew University of Jerusalem, Jerusalem 91904, Israel; ^18^Gatsby Computational Neuroscience Unit, University College London, London W1T 4JG, United Kingdom

## Abstract

Neuroscientists are increasingly initiating large-scale collaborations which bring together tens to hundreds of researchers. At this scale, such projects can tackle big challenges and engage diverse participants. Inspired by projects in mathematics, we set out to test the feasibility of widening access to such projects even further, by running a massively collaborative project in computational neuroscience. The key difference, with prior neuroscientific efforts, being that our entire project (code, results, and writing) was public from the outset, and that anyone could participate. To achieve this, we launched a public Git repository, with code for training spiking neural networks to solve a sound localization task via surrogate gradient descent. We then invited anyone, anywhere to use this code as a springboard for exploring questions of interest to them, and encouraged participants to share their work both asynchronously through Git and synchronously at online workshops. Our hope was that the resulting range of participants would allow us to make discoveries that a single team would have been unlikely to find. At a scientific level, our work investigated how a range of biological parameters, from time delays to membrane time constants and levels of inhibition, could impact sound localization in networks of spiking units. At a more macro-level, our project brought together researchers from multiple countries, provided hands-on research experience to early career participants and opportunities for supervision and teaching to later career participants. While our scientific results were not groundbreaking, our project demonstrates the potential for massively collaborative projects to transform neuroscience.

## Significance Statement

How should we structure large-scale scientific efforts? Massively collaborative projects, which anyone, anywhere, can contribute to, are one option. We ran a computational neuroscience project like this for 2 years and, here, share our results and experiences. At a scientific level, our work investigated how networks of simulated neurons can localize sound. At a more macro-level, our project brought together 31 researchers from multiple countries and provided research and training opportunities. Overall, our work demonstrates the potential for massively collaborative projects to transform how science is structured.

## Introduction

Inspired by the success of endeavors like the Human Genome Project and CERN, neuroscientists are increasingly initiating large-scale collaborations. The largest efforts, such as the International Brain Laboratory (Abbott et al., 2017; Wool, 2020), The Blue Brain Project, and Human Brain Project, bring together tens to hundreds of researchers across multiple laboratories. In terms of organization, these projects follow a formal collaborative model with open outputs. That is, there are participating laboratories who collaborate together and then make their data, methods, and results available. In this way, these projects have generated scientific insights, large-scale datasets, tools, and educational materials. But, could there be advantages to these projects being organized differently and if so what are the alternatives (Mainen et al., 2016)?

One alternative is bench marking contests, in which participants compete to obtain the best score on a specific task. Bench marking contests have driven progress in fields from computer vision (Deng et al., 2009 ) to protein folding, and have begun to enter neuroscience. For example, in Brain-Score (Schrimpf et al., 2018, 2020), participants submit models, capable of completing a visual processing task, which are then ranked according to a quantitative metric. As participants can compete both remotely and independently, these contests offer a low barrier to entry. However, defining quantifiable endpoints for neuroscientific questions remains challenging (Bowers et al., 2022).

Another alternative is massively collaborative projects, in which a problem is openly stated and contributions are welcomed from anyone willing to participate. For example, in the Polymath Project, unsolved mathematical problems are posed, and then participants share comments, ideas, and equations online as they collectively work toward solutions. Similarly, the Busy Beaver Challenge recently announced a formal proof of a conjecture that was open for decades, based mainly on online contributions from amateur mathematicians.

Inspired by this approach, we founded Collaborative Modeling of the Brain (COMOB) to explore if and how this collaborative model could be leveraged in neuroscience. This approach has two key differences from prior collaborative efforts in neuroscience. First, everything related to the project (code, results, and writing) would be openly available from the outset. As such, we could benefit from continual feedback from the community. Second, anyone, anywhere in the world would be able to participate. Meaning that individuals who may not normally have the opportunity to contribute to science would be able to do so (Mainen et al., 2016).

Here, we share our experiences and results. We start by detailing how we ran the project in terms of organization and infrastructure in “Toward Open Collaborative Science.” We then briefly summarize our scientific results in “Training SNNs for Sound Localization.” We conclude the main text with a “Discussion” of what went well, what went wrong, and how future projects like this could learn from our experiences. Finally, in “Appendices,” we provide detailed write-ups of our scientific results.

## Toward Open Collaborative Science

### Workflow

Participants joining the project were encouraged to review the material from a tutorial we ran (Goodman et al., 2022), and then to work through an introductory Jupyter Notebook (Kluyver et al., 2016) containing Python code, figures, and markdown text, which taught them how to train a spiking neural network (SNN) to perform a sound localization task. Participants were then directed to our website where we maintained a list of open scientific and technical questions for inspiration. For example, how does the use of different neuron models impact network performance and can we learn input delays with gradient descent? Then, with a proposed or novel question in hand, participants were free to approach their work as they wished. In practice, much like a “typical” research project, most work was conducted individually, shared at monthly online meetings, and then iteratively improved upon. For example, several early career researchers tackled questions full-time as their dissertation or thesis work and benefited from external input at monthly workshops.

We consciously decided on this free-form structure to experiment with the feasibility of a more bottom-up approach to doing team science, with minimal top-down supervision. We discuss the advantages and disadvantages of this approach in “Discussion.”

### Infrastructure

#### Code

**Figure 1.**
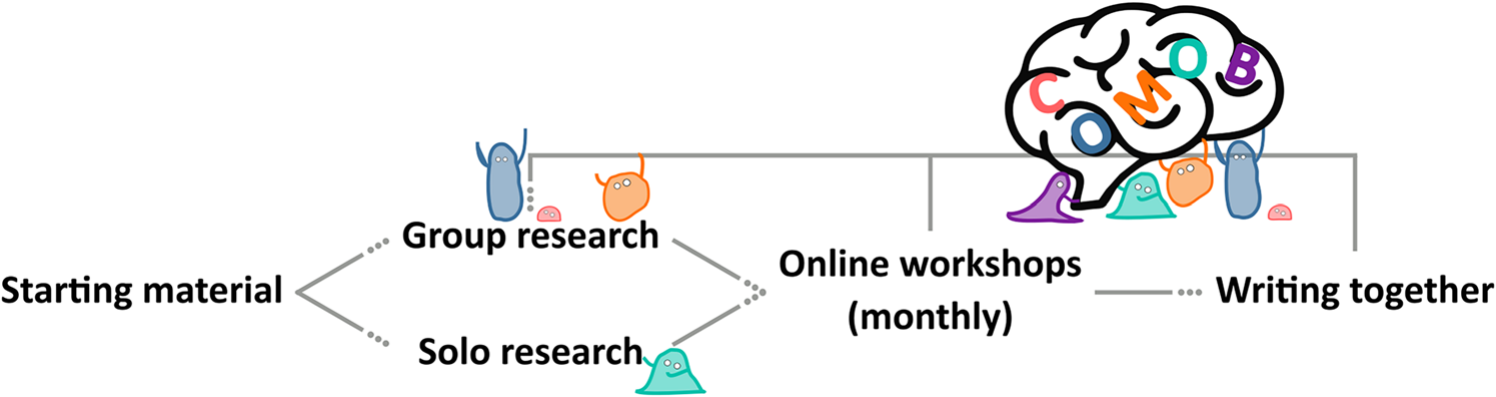
Our workflow. To on-board new participants, we provided text, videos, and code outlining our scientific starting point. This material formed a springboard for participants to pursue research either individually or in small groups. We then iteratively improved on this work through monthly online workshops and by writing this paper together through an open, collaborative process.

We provided a starting notebook to give participants an easy way to get started. This is a Python-based Jupyter Notebook (Kluyver et al., 2016), an interactive cell-based environment which allows a mixture of text, image, and code cells to be weaved together, combined with an easy user interface. Participants could either work locally using their own Python distribution or using a cloud compute service such as Google Colab. We choose Google Colab to minimize the entry barrier for participation, as it is a free service (although blocked in certain countries unfortunately) where all the software packages needed are pre-installed, meaning it takes users only a few seconds to go from reading about the project to running the code (Fig. 1).

Our starting notebook used a combination of NumPy (Harris et al., 2020), Matplotlib (Hunter, 2007), and PyTorch (Paszke et al., 2019). The code for surrogate gradient descent was based on Friedemann Zenke’s SPyTorch tutorial (Zenke and Ganguli, 2018; Zenke, 2019).

Note that we did not require participants to use our starting notebook, and indeed in “Contralateral glycinergic inhibition as a key factor in creating ITD sensitivity,” two authors implemented a very different sound localization model from scratch.

#### GitHub

Like many open-source efforts, our public GitHub repository was the heart of our project. This provided us with three main benefits. First, it made joining the project as simple as cloning and committing to the repository. Second, it allowed us to collaborate asynchronously. That is, we could easily complete work in our own time, and then share it with the group later. Third, it allowed us to track contributions to the project. Measured in this way, 28 individuals contributed to the project. However, interpreting this number is challenging, as these contributions vary significantly in size, and participants who worked in pairs or small groups often contributed under a single username. We return to this point in “Discussion.”

#### Website via MyST markdown

For those interested in pursuing a similar project, our repository can easily be used as a template. It consists of a collection of documents written in Markdown and executable Jupyter Notebooks (Kluyver et al., 2016) containing all the code for the project. Each time the repository is updated, GitHub automatically builds these documents and notebooks into a website so that the current state of the project can be seen by simply navigating to the project website. We used MyST Markdown to automate this process with minimal effort. This paper itself was written using these tools and was publicly visible throughout the project write-up.

### Teaching with this framework

This project emerged from a tutorial, and, although the project was primarily research-oriented, our code is well suited for teaching concepts from across neuroscience. As such, we integrated our project into a Physics MSc course on Biophysics and Neural Circuits. Working individually or in pairs, students actively engaged by adjusting network parameters and modifying the provided code to test their own hypotheses. Later, brief progress report presentations stimulated dynamic discussions in class, as all students, while working on the same project and code, pursued different hypotheses. We found that this setup naturally piqued interest in their peers’ presentations, enhanced their understanding of various project applications, and facilitated collaborative learning. Moreover, it allowed for engagement from students at a range of skill levels and helped bridge the gap between teaching and research. For those interested in teaching with this framework, introductory slides and dedicated Python notebooks are available on our GitHub repository.

## Training SNNs for Sound Localization

### Introduction

Animals localize sounds by detecting location- or direction-specific cues in the signals that arrive at their ears. Some of the most important sources of cues (although not the only ones) come from differences in the signals between two ears, including both level and timing differences. Respectively, termed interaural level difference (ILD) and interaural timing difference (ITD). In some cases, humans are able to detect arrival time differences as small as 20 μs.

The classic model of ITD sensitivity is the delay line model of Jeffress (1948) in which an array of binaural coincidence detector neurons receive inputs from the two ears with different delays. When a neuron’s delays exactly match the acoustic delays induced by the sound location, it will be maximally active. Therefore, the identity of the most active neuron indicates the direction of the sound. This model is widely accepted, though was shown to be inefficient with respect to neural noise by McAlpine and Grothe (2003), who proposed an alternative model based on the two binaural hemispheres average firing rates—which is optimally robust to neural noise. However, Goodman et al. (2013) showed that these models perform too poorly to account for behavioral data, especially in situations where sounds had complex and unknown spectral properties or in the presence of background noise, and proposed an alternative based on a perceptron-like neural network—which is both robust to neural noise and performed well across a range of conditions.

Building on this literature and a tutorial we ran (Goodman et al., 2022), the starting point of our project was to ask: what solutions would you find if you directly optimized a SNN to localize sounds? How would those solutions depend on the available neural mechanisms and statistics of the sound? Could we understand the solutions found? What properties would the solution have in terms of robustness to noise, generalization, and so forth? And could the solutions found by optimization throw light on features found in the auditory systems of different animals?

Two things are worth noting here. First, our solutions may be very different to optimal solutions or other neural network-based solutions. This literature is reviewed in Grumiaux et al. (2022), including classical engineering approaches, such as beamforming, and deep learning approaches, such as convolutional neural networks (CNNs), recurrent neural networks, and attention-based networks. Second, our setup is fairly limited in terms of the available cues and network structure: we only use pure tones, we have no spectral or cross-frequency cues, we fix the level so we have no ILDs, etc. We would not necessarily expect this approach to explain a wide range of observed phenomena, but it may still throw light on some fundamental aspects of interaural time or phase difference circuits.

### Materials and methods

**Figure 2.**
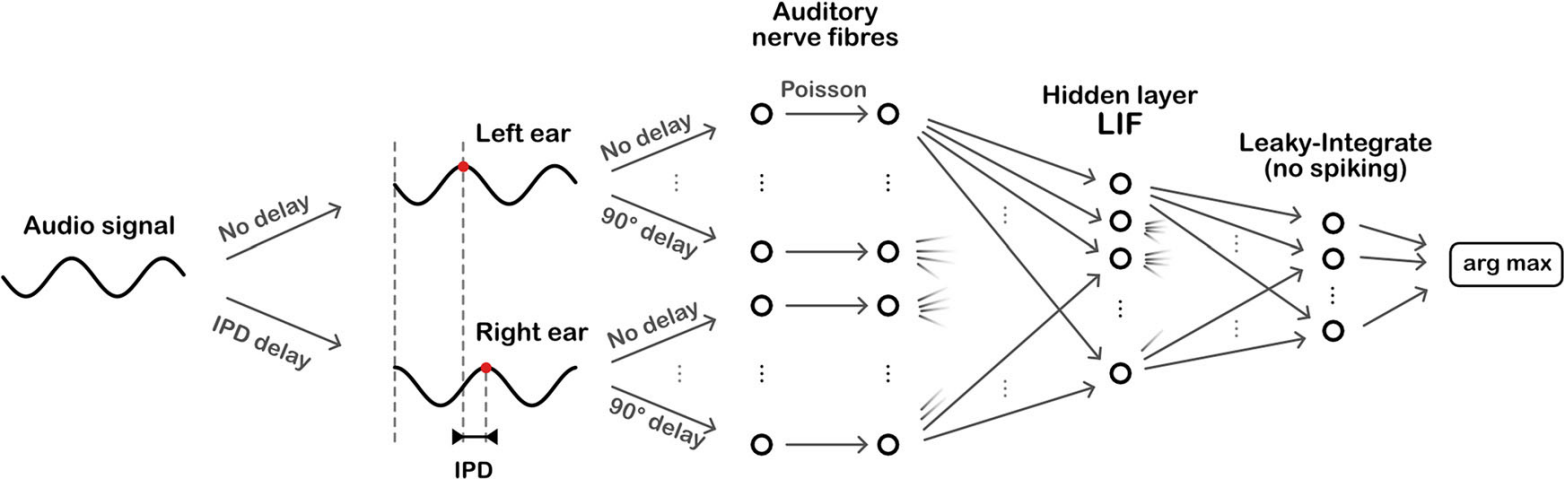
Overall model architecture. Sinusoidal audio signals are passed through two populations of units, with a range of preassigned phase delays, representing the left and right ears. These units generate Poisson spike trains which pass forward to a layer of LIF units, then a layer of leaky-integrator output units from which we readout the networks estimate of the IPD.

We started with a simple, SNN model (described in more detail in “Methods”) trained to solve a highly abstracted sound localization task (Fig. 2).

The task is to estimate the interaural phase difference (IPD) of a pure tone (sine wave) at a fixed frequency. This is equivalent to estimating the ITD, as the ITD is ambiguous for a sine wave. The model consists of three layers. First, a layer of spiking input neurons which fire spikes according to a Poisson process with a time-varying rate determined by the input stimulus, a simple model of the auditory nerve. This layer is divided into two subpopulations corresponding to the two ears, with signals to one ear delayed with respect to the other. Each neuron within a subpopulation has a different phase delay. Next comes a single hidden layer of leaky integrate-and-fire (LIF) neurons and an output layer of leaky, non-spiking neurons. Each output neuron is associated to a particular IPD, and the estimated IPD of the model is the identity of the most active output neuron.

The input neurons are all-to-all connected to the layer of hidden neurons via a trainable weight matrix. In this way, during training, the model is free to *select* the neurons with the appropriate phase delays to generate the desired properties for the hidden layer neurons. This lets the model learn to make use of delays without having to directly implement trainable delays, as this is a challenging problem (which we tackled later in “Learning delays”).

### Results

Using this setup, we successfully trained SNNs on this task and found that accuracy increased as we reduced the membrane time constant of the units in the hidden layer (Improving Performance: Optimizing the membrane time constant). This initially suggested that coincidence detection played an important role. However, further analysis (described in more detail in “A minimal trainable model of IPD processing”) showed that in fact, the network was not using a coincidence detection strategy or indeed a spike timing strategy. Rather, it appears to be using an approach similar to the equalisation-cancellation theory (Durlach, 1963; Culling, 2020) by subtracting various pairs of signals to find the point where they approximately cancel. Careful analysis of the trained model showed that it could be extremely well approximated by a six-parameter model that is quite easy to describe, but does not obviously correspond to any known features of the auditory system.

As an alternative approach, we also used tensor component analysis (TCA) (Williams et al., 2018) to explore the spiking activity of this model and to compare it across multiple trained networks (details in Tensor component analysis).

Building on this base model, we explored two main questions: how changing the neuron model alters the network’s behavior and how the phase delays (within each ear) can be learned.

#### Alternative neuron models

##### Dale’s principle

In biological networks, most neurons release the same set of transmitters from all of their synapses, and so can broadly be considered to be excitatory or inhibitory to their postsynaptic partners, a phenomenon known as Dale’s principle (Dale, 1935; Strata and Harvey, 1999). In contrast, most neural network models, including our base model, allow single units to have both positive and negative output weights.

To test the impact of restricting units to being either excitatory or inhibitory, we trained our base model across a range of inhibitory:excitatory unit ratios and tested its performance on unseen, test data (Sound localisation following Dale’ law). We found that networks which balanced excitation and inhibition performed significantly better than both inhibition-only networks, which perform at the chance level as no spikes propagate forward, and excitation-only networks, which were roughly 30% less accurate than balanced networks.

To understand where the network inhibition is required, we then trained a second set of networks in which we forced either the input or hidden units to be all excitatory and set the remaining units to be half inhibitory and half excitatory. Networks with all excitatory hidden units performed as well as networks with balanced units, while networks with purely excitatory inputs performed significantly worse, demonstrating a role for inhibition in the input-hidden connections/delay lines.

Inhibition therefore plays an important role in this model, in line with experimental data that show that blocking inhibition eliminates ITD sensitivity in the medial superior olive (MSO; Brand et al., 2002; Pecka et al., 2008).

##### Filter-and-fire

Unlike most point neuron models, in which pairs are connected by a single weight, many biological neurons make multiple contacts with their postsynaptic partners at different points along their dendritic tree. These contacts evoke postsynaptic potentials (PSPs) with distinct temporal dynamics, depending on their distance from the soma, with distal/proximal contacts inducing prolonged/brief PSPs. These features are captured by the filter-and-fire neuron model (F&F; Beniaguev et al., 2024), in which units make multiple contacts with their partners and each input is convolved with a distance-from-soma dependent synaptic filter. While networks of F&F units outperform networks of LIF units on a temporal version of MNIST, we hypothesized that this difference would be magnified in our sound localization task, given its natural temporal structure. We found that while training performance was increased using the F&F model, test performance was much worse, suggesting overfitting.

#### Learning delays

As in our base model, many studies incorporate axonal and/or dendritic delays as non-learnable parameters. Here, we explore how these phase delays, as well as synaptic delays, can be learned through two approaches.

The first method was to develop a differentiable delay layer (DDL). This method uses a combination of translation and interpolation, where the interpolation allows the delays to be differentiable even though time steps are discrete. This can be placed between any two layers in a SNN and is capable of solving the task without weight training. This work is described in more detail in “Learning delays.”

While we were developing our DDL-based method, a paper introducing synaptic delays using dilated convolutions with learnable spacings (DCLS) was published (Khalfaoui-Hassani et al., 2023b; Hammouamri et al., 2024 ), prompting us to explore this approach as well. This method also relies on interpolation and is very similar to the DDL method, serving as a generalization for synaptic delays. It uses a 1D convolution through time to simulate delays between consecutive layers. The kernels include a single non-zero weight per synapse, which corresponds to the desired delay. This method co-trains weights and delays.

We found that both methods performed well and eliminated the artificial phase delays introduced in the basic model, although DCLS reached a lower level of error overall.

#### Detailed inhibition-based model

Finally, we developed a more detailed model in which we used over 170,000 units, with conductance-based synapses, to approximate the structure of the mammalian brainstem circuit (see more details in “Contralateral glycinergic inhibition as a key factor in creating ITD sensitivity”).

In short, input spectrograms representing sounds at azimuth angles from −90° to +90° were converted into spikes, then passed forward to populations representing the globular and spherical bushy cells (GBC and SBC), and subsequently the lateral and medial superior olivary nuclei, from which we readout sound source angle predictions. Note that unlike the work with our base model, we used no learnable parameters in this model, and instead based parameters on neurophysiological data. For example, the MSO units had excitatory inputs from both the ipsi and contralateral SBCs and dominant inhibition from contralateral GBCs.

This model generated realistic tuning curves for lateral superior olive (LSO) and MSO neurons. Moreover, removing inhibition shifted ITD sensitivity to the midline, as in Brand et al. (2002) and Pecka et al. (2008).

## Discussion

### What went well

Starting from a tutorial we ran, Goodman et al. (2022), meant that users had a clear entry point to the project without needing prior expertise and a code base that was designed to be easy to understand. In addition, the popularity of the tutorial (over 38k views on YouTube at the time of writing) meant that many people heard about this project and were interested in participating. In addition, the GitHub-based infrastructure, which automatically updated our website when anyone made a change to their code or to the text of the paper, allowed for easy sharing of results.

By providing models which used spiking neurons to transform sensory inputs into behavioral outputs, participants were free to explore in virtually any direction they wished, much like an open-world or sandbox video game. Indeed, over the course of the project, we explored the full sensory-motor transformation from manipulating the nature of the input signals to perturbing unit activity and assessing network behavior. Consequently, in addition to its role in research, our code forms an excellent basis for teaching, as concepts from across neuroscience can be introduced and then implemented in class. In this direction, we integrated our project into two university courses and provide slides and a highly annotated python notebook, for those interested in teaching with these models.

Beyond providing teaching and hands-on research experience, the project also offered many opportunities for participants to improve their “soft” scientific skills. For early career researchers (undergraduate and master’s students), these included learning how to work with Git, collaborate with researchers from diverse countries and career stages, and contribute to a scientific publication. For later career researchers (PhD, Postdoc), the project provided many supervision and leadership opportunities. For example, during online workshops, later career participants were encouraged to lead small groups focused on tackling specific questions.

### What went wrong

While our sandbox design offered several advantages (discussed above), the open nature of the process did present three challenges. Our first challenge was standardizing work across participants, for example, ensuring that everyone used the same code and hyperparameters. Along these lines, future projects would benefit from having participants dedicated to maintaining the code base and standardizing participants work.

Our second challenge was the project’s exploratory nature. While this appealed to many participants, the lack of a clear goal or end-point may have been off-putting to others. For future efforts, one alternative would be to define clear goals *a priori*, however if handled carelessly, this runs the risk of reducing to a to-do list passed from more senior to more junior researchers. A more appealing alternative could be to structure the project in clearly defined phases. For example, early months reviewing literature could be followed by a period of proposals and question refinement, before a final stretch of research. Another alternative would be to begin by collecting project proposals and allowing participants to vote on a project to pursue. This could be done by having each participant check a box for each project they would choose to work on if it were selected, and then selecting the project with the most checks (thereby maximizing the expected number of participants). Multiple projects could even be launched simultaneously if this approach proved popular.

A third challenge, which arose toward the end of the project, was how to fairly assign credit. We had initially—and perhaps somewhat idealistically—stated that anyone who contributed to the project, either by writing code or participating in one of the workshops, would be included on the author list. To the extent that it was possible, we have followed through with this, though we were simply unable to contact several of the participants and so could not include them as authors. Another issue with this system is that participants with unequal contributions, e.g., attending a workshop versus contributing an entire section of the paper, would be assigned similar credit, i.e., authorship. To resolve this, we experimented with using the number or size of GitHub commits to order authors, however we found that these metrics did not accurately reflect contributions. For example, it may be quicker to commit a large-amount of low quality text than a concise well written section, and similarly there is no good reason to distinguish between two authors who submit the same amount of work through a different number of commits. We attempted to address this challenge by providing a contribution table (Contributors; Table 1) and agreeing an author order. This order was agreed on unanimously, though could easily cause issues in other projects. Consequently, we recommend that a strategy for credit assignment be determined collaboratively at the start of the project and made explicit so that participants can clearly understand how their contribution will translate to credit. Alternatively, such projects could publish under a pseudonym, e.g., COMOB.

**Table 1.** Contributors, ordered by GitHub commits as of July 16, 2024

Name	GitHub	Contribution
Tomas Fiers	@tfiers	Built the website infrastructure and created Figure 2 based on Dan’s sketch of the model architecture.
Dan Goodman	@thesamovar	Conceived the project, wrote the paper, and wrote and recorded the Cosyne tutorial. Conducted research (Starting Notebook, Analysing performance and solutions as time constants change).
Marcus Ghosh	@ghoshm	Managed the project, wrote the paper, conducted research (Quick Start Notebook, Sound localisation following Dale’ law), and gave the Cosyne tutorial
Francesco De Santis	@francescodesantis	Conducted research (Inhibition Model Notebook) and wrote the paper (“Contralateral glycinergic inhibition as a key factor in creating ITD sensitivity”)
Dilay Fidan Erçelik	@dilayercelik	Conducted research (Quick Start Notebook, Quick Start Notebook with 250 Hz input)
Pietro Monticone	@pitmonticone	Cleaned paper and notebooks
Karim Habashy	@KarimHabashy	Conducted research (Learning delays, Learning delays (v2), Vanilla sound localization problem with a single delay layer (non-spiking)), wrote the paper (“Learning delays”), and project management (Quick Start Notebook)
Balázs Mészáros	@mbalazs98	Wrote the paper (DCLS-based delay learning in the appendix) and conducted research (Noise offsets in every iteration, Dilated Convolution with Learnable Spacings)
Mingxuan Hong	@mxhong	Conducted research (Altering Output Neurons, Dynamic threshold)
Rory Byrne	@rorybyrne	Organized the source code structure and conducted research (Improving Performance: Optimizing the membrane time constant)
Sara Evers	@saraevers	Conducted research (Analysing Dale’s law and distribution of excitatory and inhibitory neurons)
Zach Friedenberger	@ZachFriedenberger	Conducted research (Improving Performance: Optimizing the membrane time constant)
Helena Yuhan Liu	@Helena-Yuhan-Liu	Conducted research (Analysis: thresholding W1W2 plot)
Jose Gomes (Portugal, PhD)	@JoseGomesJPG	Conducted research (Sound localisation following Dale’ law)
(Unknown)	@a-dtk	Conducted research (Robustness to Noise and Dropout)
Ido Aizenbud	@ido4848	Conducted research (Filter-and-Fire Neuron Model)
Sebastian Schmitt	@schmitts	Conducted research (background on neuromorphic hardware in Background)
Rowan Cockett	@rowanc1	MyST technical support
Alberto Antonietti	@alberto-antonietti	Supervised Francesco De Santis and wrote the paper (“Contralateral glycinergic inhibition as a key factor in creating ITD sensitivity”)
Juan Luis Riquelme	@luis-rr	Conducted research (Sound localisation with excitatory-only inputs surrogate gradient descent)
Adam Haber	@adamhaber	Conducted research (Compute hessians (jax version))
Gabriel Béna	@GabrielBena	Conducted research (Analysing trained networks - workshop edition, Sound localisation following Dale’ law)
Peter Crowe	@pfcrowe	Conducted research (Improving Performance: Optimizing the membrane time constant)
Umar Abubacar	@UmarAbubacar	Conducted research (TCA Analysis) and wrote the paper (“Tensor component analysis”)
Gabryel Mason-Williams	None/unknown	Conducted research (Analysing trained networks - workshop edition)
Josh Bourne	None/unknown	Conducted research (Analysing trained networks - workshop edition)
Zekai Xu	None/unknown	Conducted research (Analysing trained networks - workshop edition)
Leonidas Richter	None/unknown	Conducted research (Learning delays)
Chen Li	None/unknown	Conducted research (Improving Performance: Optimizing the membrane time constant)
Brendan Bicknell	None/unknown	Supervised Dilay Fidan Erçelik
Volker Bormuth	None/unknown	Developed teaching materials and used the project to teach two university courses. Supervised Marcus Ghosh and students at Sorbonne University.

Ultimately, while the project explored many interesting directions, which will form the basis for future work, we did not reach a point where we could draw strong scientific conclusions about sound localization. From group discussions, we concluded that this is likely due to the free-form nature of our project, which would have benefited from a more coordinated approach. The question is, how to do this without compromising the ideals of a grass-roots project? Extending the voting idea above, one approach would be to make the proposer of the democratically selected project responsible for making sure that results are comparable and generally keeping the project on the right track. A role similar to a traditional supervisor, but with the critical difference that they are elected by their peers and only on a project by project basis.

### Conclusions

This paper does not present a scientific breakthrough. However, it does demonstrate the feasibility of open research projects which bring together a large number of participants across countries and career stages to work together collaboratively on scientific projects. Looking ahead, we hope that by lowering the barrier to entry, these projects will welcome a wider and more diverse set of expertise and perspectives, generate new ideas, and lead to discoveries beyond what any single group could realize.

## Notebook Map

The following lists the notebooks, authors, summary, and related notebooks in this project.

### Introductory notebooks

Explanation of the background: (author: Dan Goodman).
List of research questions and challenges: (author: everyone).

### Templates/starting points

Starting Notebook: The template notebook suggested as a starting point, based on the Cosyne tutorial that kicked off this project (author: Dan Goodman).
Quick Start Notebook: Condensed version of Starting Notebook using the shorter membrane time constants from Improving Performance: Optimizing the membrane time constant and Dale’s law from Sound localisation following Dale’ law (authors: Dilay Fidan Erçelik, Karim Habashy, and Marcus Ghosh).

### Individual notebooks

Filter-and-Fire Neuron Model: Using an alternative neuron model (author: Ido Aizenbud based on work from Dilay Fidan Erçelik).
Altering Output Neurons: Comparison of three different ways of reading out the network’s decision (average membrane potential, maximum membrane potential, and spiking outputs) with short and long time constants (author: Mingxuan Hong).
Analysing trained networks—workshop edition: Group project from an early workshop looking at hidden unit spiking activity and single unit ablations. Found that some hidden neurons do not spike, and ablating those does not harm performance. Builds on (WIP) Analysing trained networks (authors: Gabriel Béna, Josh Bourne, Tomas Fiers, Tanushri Kabra, and Zekai Xu).
Sound localisation following Dale’ law: Investigation into the results of imposing Dale’s law. Incorporated into Quick Start Notebook. Uses a fix from Analysing Dale’s law and distribution of excitatory and inhibitory neurons (authors: Marcus Ghosh, Gabriel Béna, and Jose Gomes).
Dynamic threshold: Adds an adaptive threshold to the neuron model and compares results. Conclusion is that the dynamic threshold does not help in this case (author: Mingxuan Hong).
Sound localisation with excitatory-only inputs surrogate gradient descent: Results of imposing an excitatory-only constraint on the neurons. Appears to find solutions that are more like what would be expected from the Jeffress model (author: Juan Luis Riquelme).
Learning delays, Learning delays (v2) and Vanilla sound localization problem with a single delay layer (non-spiking), Delay learning using DDL, written up in “Learning delays” (author: Karim Habashy).
Dilated Convolution with Learnable Spacings: Delay learning using dilated convolution with learnable spacings, written up in “Learning delays” (author: Balázs Mészáros).
Robustness to Noise and Dropout: Tests effect of adding Gaussian noise and/or dropout during training phase. Conclusion is that dropout does not help and adding noise decreases performance (author: Unknown (@a-dtk)).
Version with 250 Hz input, Quick Start Notebook with 250 Hz input: Analysis of results with a higher frequency input stimulus and different membrane time constants for hidden and output layers. Conclusion is that smaller time constant matters for hidden layer but not for output layer (author: Dilay Fidan Erçelik).
Analysing performance and solutions as time constants change: Deeper analysis of strategies found by trained networks as time constants vary. Added firing rate regularization. Extends Improving Performance: Optimizing the membrane time constant. Written up in more detail in “A minimal trainable model of IPD processing” (author: Dan Goodman).
Workshop 1 Write-up Write-up of what happened at the first workshop: (author: Marcus Ghosh).

### Inconclusive

The following notebooks did not reach a solid conclusion.

Compute hessians (jax version): An unfinished attempt to perform sensitivity analysis using Hessian matrices computed via autodifferentiation with the Jax library (author: Adam Haber).
Analysis of an alternative way of handling noise: (author: Balázs Mészáros).
Unfinished attempt to improve analysis code: (author: Helena Yuhan Liu).

### Historical

This subsection includes notebooks whose content got merged into an updated notebook later.

(WIP) Analysing trained networks: Early work on analyzing the strategies learned by trained networks. Folded into Analysing trained networks—workshop edition (author: Dan Goodman).
Improving Performance: Optimizing the membrane time constant: Analyzes how performance depends on membrane time constant. Folded into Analysing performance and solutions as time constants change (authors: Zach Friedenberger, Chen Li, and Peter Crowe).
Analysing Dale’s law and distribution of excitatory and inhibitory neurons: Fixed a mistake in an earlier version of Sound localisation following Dale’ law (author: Sara Evers).

## Appendices

In this section, we provide detailed write-ups of our scientific results.

### A minimal trainable model of IPD processing

**Table:** 

Section authors	Dan Goodman
Notebooks	Analysing performance and solutions as time constants change

This section describes the initial model developed for the Cosyne tutorial that served as the starting point for this project (Goodman et al., 2022). It also describes some small variants of this basic model produced in the course of the project, which can be seen in the notebook Analysing performance and solutions as time constants change. The background and aims of this model are described in “Introduction.”

#### Methods

The model consists of the following pathway, illustrated in Figure 2: IPD → stimulus → input neurons → hidden layer neurons → readout neurons.

The IPD is an angle uniformly randomly selected in *α* ∈ [ − *π*/2, *π*/2] (frontal plane only).

The stimulus is a sine wave, and we model IPDs by adding the IPD *α* as a phase delay to the right ear. Enumerating the ears with index *i* ∈ {0, 1} so that *i* = 0 is the left ear, we get that the stimulus in ear *i* is

$$S_{i}(t)=\sin\;(2\pi ft+i\alpha )$$

To model a population of spiking neurons with different lags, we associate each input layer neuron with an ear and an additional phase delay *ψ* uniformly spaced in the range (0, *π*/2). This ensures that by comparing a left and right ear, you can generate any phase difference in the range ( − *π*/2, *π*/2) as required. Specifically, we set neuron *j* connected to ear *i* to receive the stimulus:

$$S_{ij}(t)=\sin\;(\theta_{ij})$$

where

$$\theta_{ij}(t)=2\pi ft+i\alpha +\psi_{j}$$

$$\psi_{j}=j\pi /2(N_{\psi }-1)$$

and $N_{\psi }$ is the number of input neurons per ear.

**Figure 3.**
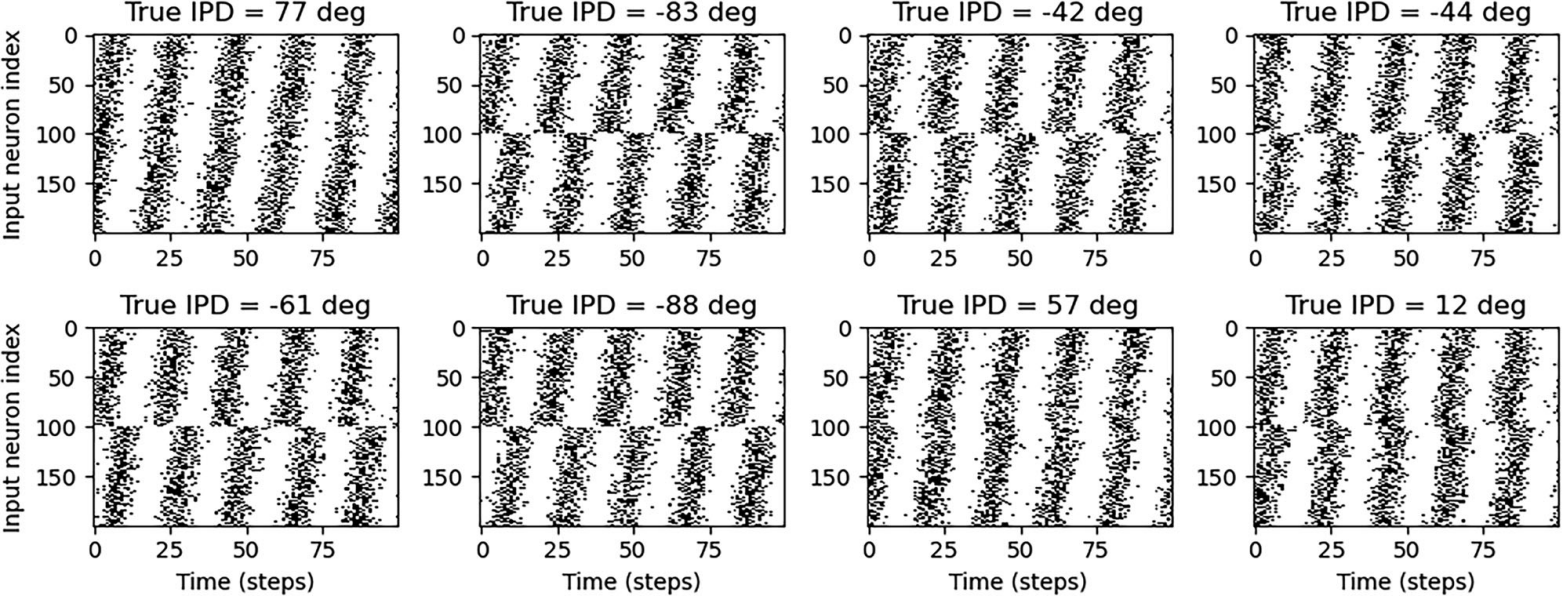
Examples of generated input spike trains. Each plot shows a raster plot of input spikes for a different sample. The subtitle of each plot gives the true interaural time difference (ITD). The *x*-axis is time in steps for the whole input duration, and the *y*-axis is input neuron index. Input neurons are in two groups, with each “ear” consisting of 100 neurons. Within each group, the spikes are delayed by an increasing amount from a minimum to maximum delay. Spikes are produced by a Poisson process with a time-varying firing rate given by a sinusoid.

Next, we make these neurons spiking by giving them a time-varying firing rate:

$$R_{ij}(t)=R_{\max}((1+\sin\;\theta_{ij}(t))/2{)}^{2}$$

where *R*_max_ is the maximum firing rate, which we set as 600 sp/s. Spikes are then generated via an inhomogeneous Poisson process with intensity *R*_*ij*_(*t*). Some example raster plots are shown in Figure 3.

The $2N_{\psi }$ input neurons are connected all-to-all to a “hidden layer” of *N*_*h*_ spiking neurons. These are standard LIF neurons with a membrane potential *v* that in the absence of spikes evolves over time according to the differential equation:

$$\tau \frac{dv}{dt}=-v$$

where *τ* is the membrane time constant. An incoming spike on a synapse with weight *w* causes an instantaneous increase *v* ← *v* + *w*. These weights are stored in a matrix *W*_*ih*_ of size $\left(2N_{\psi },N_{h}\right)$. If *v* crosses the spike threshold of 1, the neuron emits a spike and instantaneously resets to *v* ← 0.

The hidden layer is all-to-all connected to a readout layer of *N*_*c*_ neurons via a weight matrix *W*_*ho*_. The aim is to divide the set of possible IPDs into *N*_*c*_ intervals *I*_*k*_ = [ − *π*/2 + *kπ*/*N*_*c*_, − *π*/2 + (*k* + 1)*π*/*N*_*c*_] and then, if neuron *k* is the most active, guess that the IPD must be in interval *I*_*k*_. These hidden layer neurons follow the same differential equation but do not spike. Instead, to guess the IPD, we compute their mean membrane potential over the duration of the stimulus, ${\bar{v}}_{k}$, and then compute an output vector that is the log softmax function of these mean membrane potentials:

$$x_{k}=\log\;\frac{\exp\;{\bar{v}}_{k}}{\sum_{\ell }\exp\;{\bar{v}}_{\ell }}$$

We then interpret *x*_*k*_ as the estimated probability that *α* ∈ *I*_*k*_. Our estimate of the IPD $\hat{\alpha }$ will be the midpoint of the interval corresponding to the most active neuron $\hat{k}={\mathrm{argmax}}_{k}\;x_{k}$. Note that the softmax function and probability interpretation are important for training the network, but once the network is trained, you can equally well compute $\hat{k}={\mathrm{argmax}}_{k}\;{\bar{v}}_{k}$.

The network is trained by defining a loss function that increases the further away the network behavior is from what we would like (defined in detail below), and then using the surrogate gradient descent method (Zenke and Ganguli, 2018; Neftci et al., 2019). Full details on training parameters can be found in the notebook Starting Notebook.

The loss function we use is composed of two terms. The first is the cross-entropy or negative log likelihood loss that measures how far our predicted probability distribution *x*_*k*_ is from the true probability distribution (which has value 1 for the correct *k* and 0 for all other *k*). The second term, which is not used in all the notebooks in this project, is an optional regularization term. In Analysing performance and solutions as time constants change, we regularize based on the firing rates of the hidden layer neurons. We compute the firing rate for each hidden neuron *r*_*m*_. If this is below a mimimum threshold *r*_−_, it contributes nothing to the loss, otherwise we compute *L*_*m*_ = ((*r*_*m*_ − *r*_−_)/(*r*_+_ − *r*_−_))^2^ for each neuron for a constant *r*_+_ explained below. We now compute the average and multiply a constant $L=c\sum_{m}L_{m}/N_{h}$. The constant *r*_+_ is the maximum firing rate we would like to see in the network, so that *L*_*m*_ = 1 if *r*_*m*_ = *r*_+_. The constant *c* is chosen to be the expected initial cross-entropy loss of the network before training. This makes sure that a firing rate of *r*_*m*_ = *r*_+_ is heavily penalized relative to the cross-entropy loss, but that any firing rate below *r*_−_ is fine. We chose *r*_−_ = 100 sp/s and *r*_+_ = 200 sp/s.

For the results in this section, the model is trained on 128^2^ = 16, 384 samples in batches of 128, for 100 epochs using the Adam optimizer (Kingma and Ba, 2017) with a learning rate of 0.001. The network needs to be retrained for each frequency, in this section we only use *f* = 50 Hz. Test results are shown for a fresh draw of 4,096 samples.

#### Results

**Figure 4.**
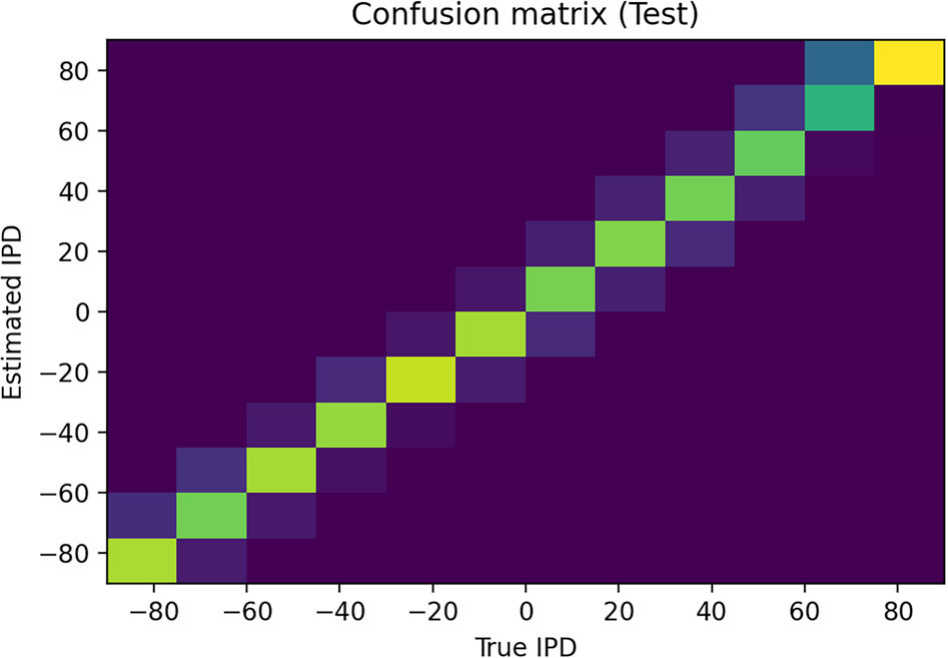
Confusion matrix. True IPD is shown on the *x*-axis, and estimated IPD on the *y*-axis. Color scale is yellow-blue, so a perfect result would be indicated by a yellow diagonal on a blue background. This plot shows the results of training the network with tone frequency *f* = 50 Hz, membrane time constant *τ* = 2 ms, number of input neurons per neuron $N_{\psi }=100$, number of hidden layer units *N*_*h*_ = 8, and number of discrete IPD categories *N*_*c*_ = 12. Mean absolute IPD errors are $∼2.6^{\circ }$.

**Figure 5.**
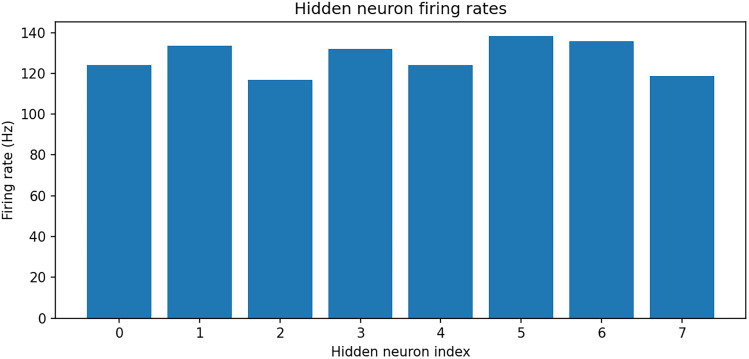
Hidden neuron firing rates, with the same setup as in Figure 4.

This approach is able to train a network that can perform the task well, using very few neurons ( $N_{\psi }=100$ input neurons per ear, *N*_*h*_ = 8 hidden neurons, and *N*_*c*_ = 12 output neurons). Mean absolute error in IPD is $∼2.6^{\circ }$ (Fig. 4). Hidden neuron firing rates are between 110 and 150 sp/s (Fig. 5).

**Figure 6.**
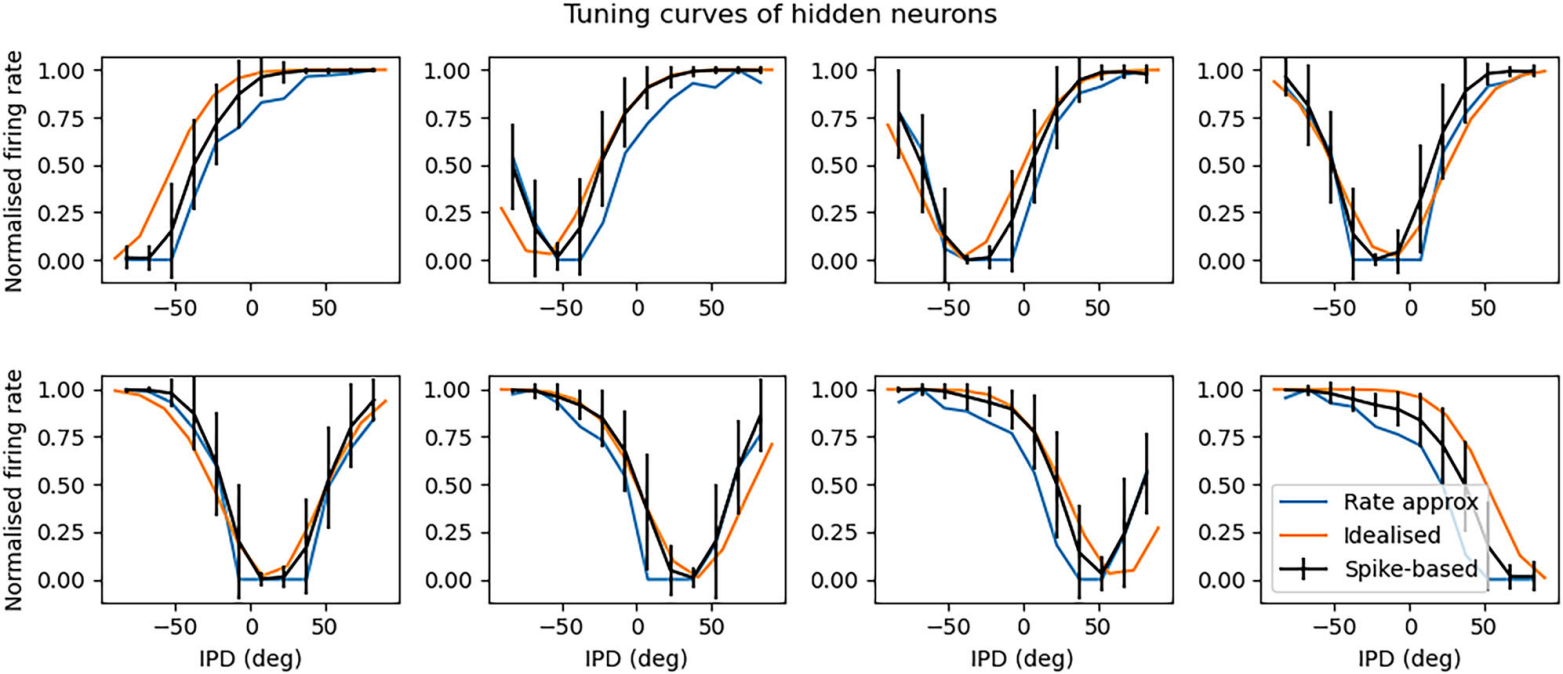
Tuning curves of hidden neurons. Each plot shows the IPD tuning curve of one of the eight hidden layer neurons in the model. The *x*-axis shows the IPD and the *y*-axis the normalized firing rate. The black curves show the results for the trained SNN. The orange curves show the best fit by a translated and scaled Gaussian curve. The blue curves show the fit for a rate-based approximation where spike times are ignored. Parameters are as in Figure 4: *f* = 50 Hz, *τ* = 2 ms, $N_{\psi }=100$, *N*_*h*_ = 8, and *N*_*c*_ = 12.

**Figure 7.**
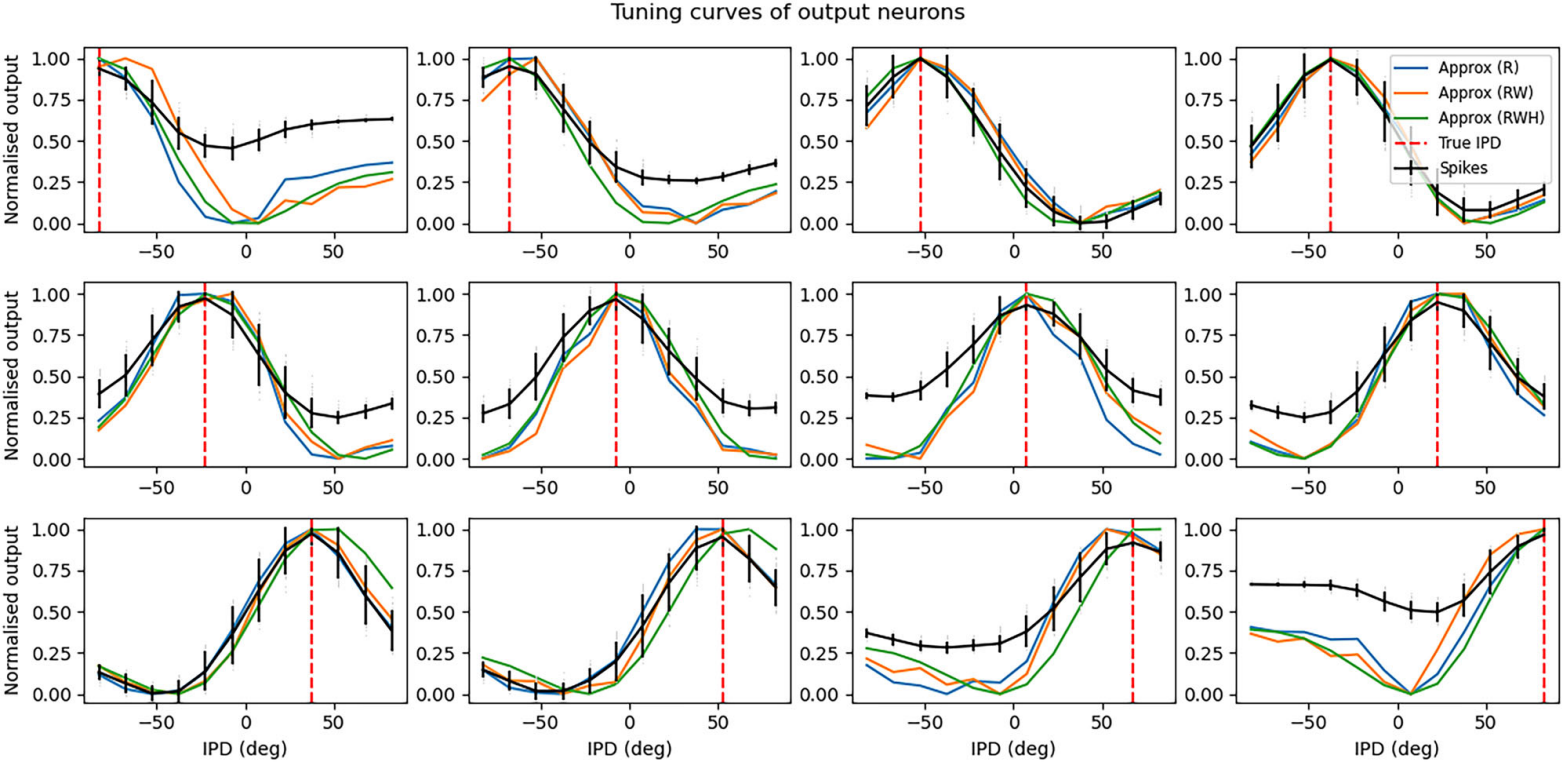
Tuning curves of output neurons. Each plot shows the IPD tuning curve of one of the eight hidden layer neurons in the model. The *x*-axis shows the IPD and the *y*-axis the normalized firing rate. The black curves show the results for the trained SNN. The blue lines show the fit with a rate-based approximation that ignores spike times. The orange lines show the results if we ignore the trained weight matrix and use the Ricker wavelet approximation described in the text. The green curve shows the tuning curves if we use both approximations, and additionally use the idealized Gaussian fits for the hidden neurons. The dashed red lines indicate the estimated IPD if that neuron is the most active. Parameters are as in Figure 4: *f* = 50 Hz, *τ* = 2 ms, $N_{\psi }=100$, *N*_*h*_ = 8, and *N*_*c*_ = 12.

**Figure 8.**
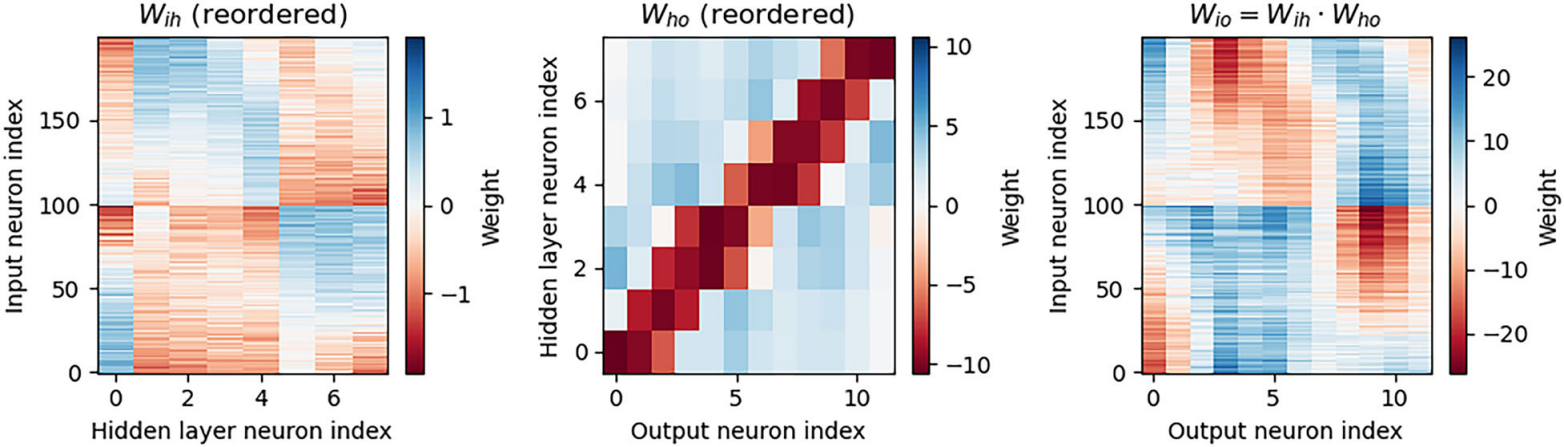
Weight matrices, with hidden neurons reordered by their worst delays. The left image shows the weight matrix from the input layer to the hidden layer. The middle image from the hidden layer to the output layer. The right image shows the product of these two, which would be the equivalent weight matrix from the input to output layers if there were no nonlinearity in the system. Colors are on a hot cold scale, with hot colors corresponding to negative weights, cold colors to positive weights, and white corresponding to zero weight. Note that the color scale is different for each image. Parameters are as in Figure 4: *f* = 50 Hz, *τ* = 2 ms, $N_{\psi }=100$, *N*_*h*_ = 8, and *N*_*c*_ = 12.

Analysis of the trained networks shows that it uses an unexpected strategy. First, the hidden layer neurons might have been expected to behave like the encoded neurons in Jeffress’ place theory, and like recordings of neurons in the auditory system, with a low baseline response and an increase for a preferred phase difference (best phase). However, very reliably they find an inverse strategy of having a high baseline response with a reduced response at a least preferred phase difference (Fig. 6). Note that the hidden layer neurons have been reordered in order of their least preferred delay to highlight this structure. These shapes are consistently learned, but the ordering is random. By contrast, the output neurons have the expected shape (Fig. 7). Interestingly, the tuning curves are much flatter at the extremes close to an IPD of ±*π*/2. We can get further insight into the strategy found by plotting the weight matrices *W*_*ih*_ from the input layer to the hidden layer, and *W*_*ho*_ from the hidden layer to the output layer, as well as their product *W*_*io*_ = *W*_*ih*_ · *W*_*ho*_ which would give the input–output matrix for a linearized version of the network (Fig. 8).

A number of features emerge from this analysis. The first is that the tuning curves of the hidden neurons have a very regular structure of having a high baseline firing rate with a dip around a “least preferred” delay that varies uniformly in the range −*π*/2 to *π*/2. Indeed, the tuning curves *i* can be very well fit with the function $a+be^{-(\alpha -\alpha_{i}{)}^{2}/2\sigma_{\alpha }^{2}}$ where *α* is the IPD, *α*_*i*_ = −*π*/2 + *iπ*/*N*_*h*_ is the “least preferred” IPD, and $a,b,\sigma_{\alpha }$ are parameters to fit (Fig. 6, orange lines). This would look likely to be consistent with some form of optimal coding theory that minimizes the effect of the Poisson noise in the spike counts, although we did not pursue this explanation.

The second feature is that spike timing does not appear to play a significant role in this network. This may initially appear surprising but in fact it is inevitable because of the coding scheme we have used where the initial layer of neurons fire Poisson spikes, and there is only one spiking layer, meaning there is no scope for spike times to be used (a limitation of this model realized late in the process). Indeed, if we predict the output of the network purely using the firing rates of the input stimulus passed through the weight matrices *W*_*ih*_ and *W*_*ho*_ plus a static nonlinearity for the input layer to the hidden layer, we get an excellent approximation for the hidden neurons (Fig. 6, blue lines) and a qualitatively good fit for the output neurons (Fig. 7, blue lines). Specifically, if *r*_*i*_(*t*) is the instantaneous time-varying firing rate of input neuron *i*, we approximate the instantaneous hidden units firing rates by the function:

$$r_{h}(t)=\left\{ \begin{array}{cc} 0 & \text{if }r_{i}(t)\leq 1\\ \frac{1}{t_{\mathrm{refrac}}+\tau \log\;(r_{i}(t)/\left(r_{i}(t)-1\right))} & \text{if }r_{i}(t)>1 \end{array}\right.$$

This function is the firing rate of a LIF neuron $\tau v^{\prime }=r_{i}(t)-v$ with a refractory period *t*_refrac_ (which is d*t* in our case because of the way it is simulated) if the function *r*_*i*_(*t*) were constant over time, but it fits well even with a time-varying *r*_*i*_(*t*). From this, we can take an average over time to get the mean firing rate. The output units do not spike so their activity is simply approximated by $r_{o}(t)=\sum_{h}W_{ho}r_{h}(t)$.

Finally, we note that the weight matrix *W*_*ho*_ visible in Figure 8 seems to have a very regular structure of weights that have a broad excitation and a narrowly tuned inhibition (an unusual pattern). Indeed, we can fit this well with a Ricker wavelet (or “Mexican hat”) function:

$$W_{ho}\approx a(1-(\delta /\sigma_{\delta }{)}^{2})e^{-\delta^{2}/2\sigma_{\delta }^{2}}+b$$

where *δ* = *o* − *N*_*c*_ *h*/*N*_*h*_, *h* and *o* are the indices of the hidden and output neurons, *N*_*h*_ is the number of hidden neurons, *N*_*c*_ is the number of output neurons, and *a*, *b*, and $\sigma_{\delta }$ are parameters to estimate. Using this approximation and the rate-based approximation from before, we get the orange curves in Figure 7. If we use both the Ricker wavelet approximation of *W*_*ho*_ and the idealized tuning curves, we get the green curves. All in all, this gives us a six-parameter model that fits the data extremely well, a significant reduction on the 896 parameters for the full model ( $N_{\psi }N_{h}+N_{h}N_{c}$).

#### Discussion

This subproject was an extension of the original notebook Starting Notebook with the aim of understanding the solutions found in more detail. We successfully found a six-parameter reduced model that behaves extremely similarly to the full model, and we can therefore say that we have largely understood the nature of this solution. We did not look in detail for a deep mathematical reason why this is the solution that is found, and this would make for an interesting follow-up. Are these tuning curves and weights Bayes optimal to reduce the effect of the Poisson spiking noise, for example?

The solution that was found gives tuning curves that are unlike those found in natural auditory systems, with an inverted “least preferred phase” structure instead of the typical “preferred phase.” In addition, the weight matrix from the hidden layer to the output layer has broadly tuned excitation and narrowly tuned inhibition, which is an unusual pattern. However, it is worth noting that the model here of detecting IPDs of sinusoids with a fixed amplitude is very simplistic compared to the real conditions faced by the auditory system.

The solution found does not appear to use coincidence detection properties of spiking neurons and indeed can be well approximated by a purely rate-based approximation. It appears to find a solution similar to that suggested by the equalisation-cancellation theory (Durlach, 1963; Culling, 2020). This seems initially surprising, but in fact because of the nature of the input stimulus (Poisson spike trains) and the fact that there is only one spiking layer of neurons, there is no temporal structure for coincidence detection to make use of, so it was inevitable that it would not find a solution that uses this strategy. An interesting follow-up would be to use a more detailed model of neuronal firing in the cochlear nucleus for example, or a multi-layer structure, and see if different solutions are found.

### Tensor component analysis

**Table:** 

Section authors	Umar Abubacar
Notebooks	TCA Analysis

To explore the spiking activity of the hidden units in our simple, neural network model, we used TCA (Williams et al., 2018). Conceptually, this method identifies groups/ensembles of neurons with similar activity patterns, termed components. More specifically, each component, identified by the algorithm, is composed of three factors:

- A neuron factor—describing how strongly associated each neuron is with each component.
- A time factor—indicating how the activity of each component changes within a trial.
- A trial factor—denoting how active each component is on each trial.

Notably, the number of components, termed the rank, is a hyperparameter which requires some consideration.

#### Methods

To acquire the necessary data, we trained the basic model and recorded the spiking activity of its hidden layer. We then smoothed each unit’s activity over time using a Gaussian kernel. Then applied nonnegative TCA using the Tensortools library.

#### Results

**Figure 9.**
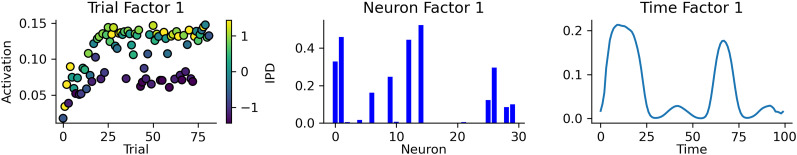
Applying TCA, with a single rank, to the spikes collected from a single neural network’s hidden layer during training. Left: this component’s activation (*y*-axis) across a subset of training trials (*x*-axis), each trial is colored by its IPD from −*π*/2 (blue) to *π*/2 (yellow). Middle: of the network’s 30 hidden units (*x*-axis) only a subset are strongly associated (*y*-axis) with this component. Right: the activity of this component (*y*-axis) over time (*x*-axis) within trials resembles a sinusoid.

As a first pass, we began by recording the spikes from a model during training and running TCA with a single rank (Fig. 9). This single component contained a subset of the hidden units (middle panel), which were more active during trials with positive IPDs (left panel) and tended to have sinusoidal-like patterns of activity (right panel).

**Figure 10.**
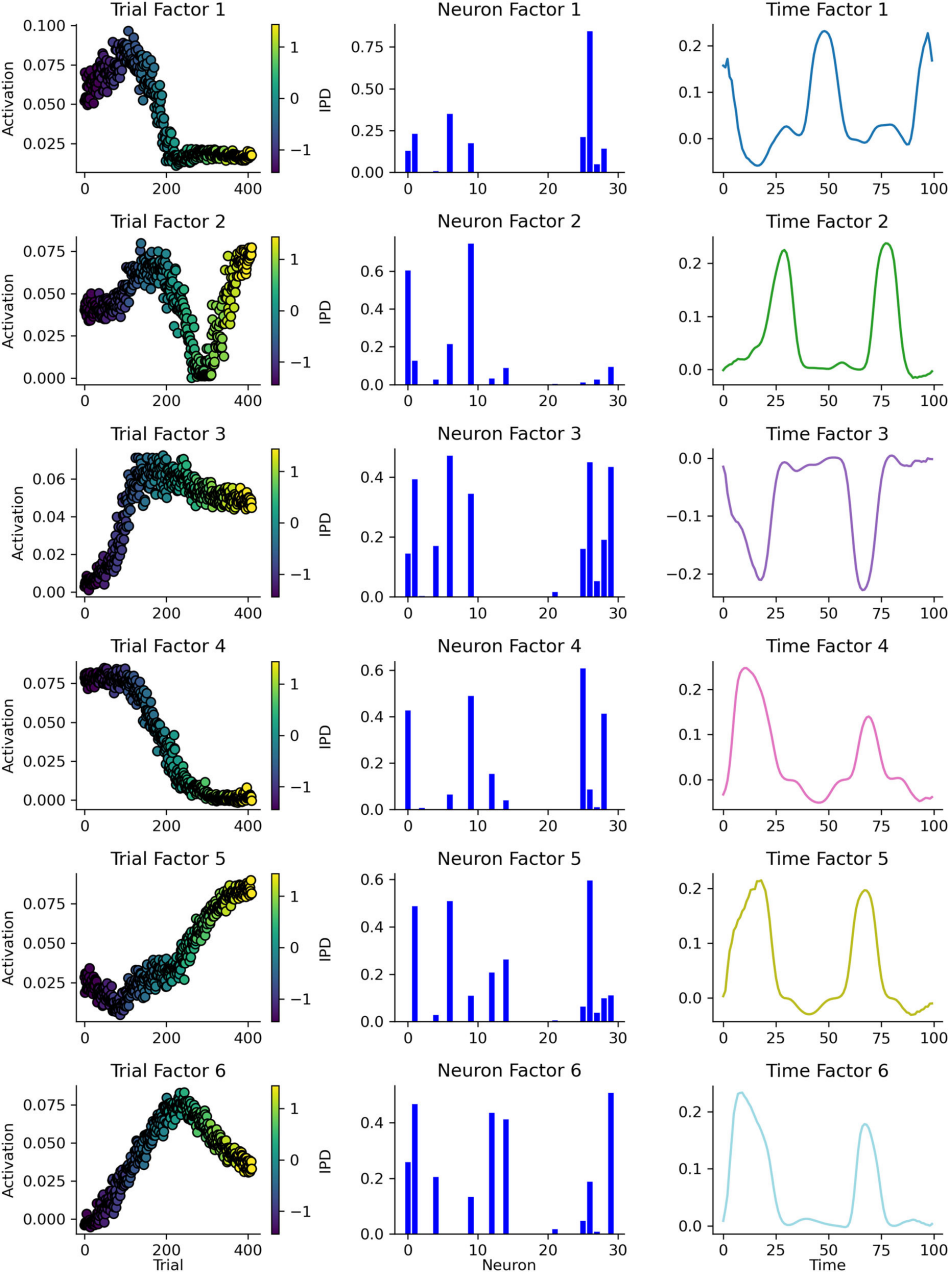
TCA analysis, with six ranks, of a trained network’s spiking in response to a range of IPDs. Each row shows one of six identified components. Left column: each components trial factor—i.e., its activation (*y*-axis) across a set of test trials (*x*-axis). Each test trial is colored by its IPD from −*π*/2 (blue) to *π*/2 (yellow). Middle column: of the network’s 30 hidden units (*x*-axis), slightly different subsets are associated with each component. Right: the activity of each component (*y*-axis) over time (*x*-axis) within trials.

Next, we took a trained network, recorded its spikes in response to a range of IPDs, and then used TCA to identify six components (Fig. 10). While some units were associated with multiple components (middle column) and all of the component’s temporal factors were generally sinusoid-like (right column), each components activity was strongly modulated by the trial’s IPD (left column). For example, component 4 was strongly active on trials with a negative IPD and virtually inactive on trials with a positive IPD. While, component 5 showed the opposite behavior. Taken together, this suggests that some of network’s hidden units are selectively responsive to particular IPD input signals.

Finally, we experimented with training multiple networks, analyzing their spiking activity with TCA and comparing the results. Our preliminary analysis of these data can be found here.

### Learning delays

**Table:** 

Section authors	Karim G. Habashy and Balázs Mészáros
Notebooks	Learning delays, Learning delays (v2), Vanilla sound localization problem with a single delay layer (non-spiking), Dilated Convolution with Learnable Spacings

We introduce a simple method to solve the sound localization problem with only learnable delays, and discuss a method that learns both weights and delays, introduced in Hammouamri et al. (2024).

#### Introduction

Following the classic work by Jeffress (1948) on axonal delays, Kempter et al. (2001) investigated the question of how ITD computation maps can arise ontogenetically in the laminar nucleus of the barn owl. They showed that ITDs computational maps emerge from the combined effect of a Hebbian spike-based learning rule and its transmission along the presynaptic axon. In other words, axonal delays shape the network structure when coupled with temporal learning rules. Based on this insight, several studies investigated the combined effect of spike timing-dependent plasticity (STDP), axonal conduction delays, and oscillatory activity on recurrent connections in spiking networks. Kerr et al. (2013) demonstrated the selective potentiation of recurrent connections in this scenario, while Kato and Ikeguchi (2016) showed that neural selection for memory formation depends on neural competition and, in turn, for neural competition to emerge in recurrent networks, spontaneously induced neural oscillation coupled with STDP and axonal delays are a prerequisite.

We can use this to develop an intuition behind coupling neuronal delays with STDP. The sign of the STDP rule depends on the order of postsynaptic and presynaptic spiking, and axonal delays can effectively reverse this. For example, the arrival of presynaptic spikes at the synapse before the backpropagated action potential leads to a synaptic depression. However, reducing the axonal transmission speed would lead to potentiation. In this line of thought, Asl et al. (2017) studied the combined role of delays and STDP on the emergent synaptic structure in neural networks. It was shown that qualitatively different connectivity patterns arise due to the interplay between axonal and dendritic delays, as the synapse and cell body can have different temporal spike orders.

Aside from their role in modeling cortical functions or shaping a network’s synaptic structure, another line of research emerged from the seminal work by Izhikevich (2006). They showed that when including conduction delays and STDP into their simulation of realistic neural models, polychronous groups of neurons emerge (although Pauli et al., 2018 suggest that some of these results may be implementation-specific artifacts). These groups show time-locked spiking patterns with millisecond precision. Subsequent studies investigated the properties and functions of such neuronal groups. For example, Szatmáry and Izhikevich (2010) demonstrated the natural emergence of large memory content and working memory when the neuronal model exploits temporal codes. Specifically, short-term plasticity can briefly strengthen the synapses of specific polychronous neuronal groups (PNGs) resulting in an enhancement of their spontaneous reactivation rates. In a qualitatively different study, Eguchi et al. (2018) showed that networks that exhibit PNG possess potential capabilities that might solve the dynamic binding problem. These networks respond with stable spatiotemporal spike trains when presented with input images in the form of randomized Poisson spike trains. The functionality of these kinds of networks emerged due to the interplay of various factors including: (i) random distribution of axonal delays, (ii) STDP, and (iii) lateral, bottom-up and top-down synaptic connections.

Finally, it should be noted that most of the studies that incorporate axonal and/or dendritic delays include them as a non-learnable parameter. Few studies investigated the possibility of training transmission delays in order to enhance the computational capabilities of SNNs. Matsubara (2017) proposed an algorithm that modifies the axonal delays and synaptic efficacy in both supervised and unsupervised approaches. The learning method involved approximates the expectation–maximization (EM) algorithm and, after training, the network learns to map spatiotemporal input–output spike patterns. Thus, EM is one way to train SNNs that are cast as probabilistic models. Another approach that exploits recent developments in deep learning is Hammouamri et al. (2024). In this work, delays are represented as 1D convolutions through time, where the kernels include a single per-synapse non-zero weight. The temporal position of these non-zero weights corresponds to the desired delays. The proposed method co-trains weights and delays and is based on the dilated convolution with learnable spacings (DCLS) algorithm (Khalfaoui-Hassani et al., 2023a).

In this work, we propose a delay learning algorithm that is simple and efficient. The delay learning is mediated by a DDL. This layer can be inserted between any two layers in an SNN in order to learn the appropriate delay to solve a machine learning task. This DDL is architecture agnostic. Also, the method is designed to learn delays separately from synaptic weights.

#### Methods

The DDL is, mainly, based on a 1D version of the spatial transformer network (STN; Jaderberg et al., 2015). The STN is a differentiable module that can be added into CNNs architectures to empower them with the ability to spatially transform feature maps in a differentiable way. This addition leads to CNN models that are invariant to various spatial transformations like translation, scaling, and rotation. Image manipulations are inherently not differentiable because pixels are discrete. However, this problem is overcome by the application of an interpolation (for example, bi-linear) after the spatial transformation.

The DDL is a 1D version of the spatial transformer where the only transformation used is translation. Translation of a spike along the time dimension can be thought of as a translation of a pixel along the spatial coordinates. The general affine transformation matrix for the 2D case takes the form in the following equation:

$$\left[\begin{array}{ccc} sr_{1} & sr_{2} & t_{x}\\ sr_{3} & sr_{4} & t_{y}\\ 0 & 0 & 1 \end{array}\right]\left[\begin{array}{c} x_{t}\\ y_{t}\\ 1 \end{array}\right]=\left[\begin{array}{c} x_{s}\\ y_{s}\\ 1 \end{array}\right]$$

In the above equation, *sr*_1_, *sr*_2_, *sr*_3_, *sr*_4_ are the elements responsible for the linear transformations of scaling, rotation, and shear. *t*_*x*_ and *t*_*y*_ are the translations in the *x*-axis and *y*-axis, respectively. *x*_*t*_ and *y*_*t*_ are the location of a spike/pixel (in case of spikes *y* = 0) in the target/output grid, while *x*_*s*_ and *y*_*s*_ are the location of the source grid. A grid can be an image or a 2D array of spike trains. For the case of only translation along the *x*-axis, the affine transformation matrix becomes

$$\left[\begin{array}{ccc} 1 & 0 & t_{x}\\ 0 & 1 & 0\\ 0 & 0 & 1 \end{array}\right]$$

**Figure 11.**
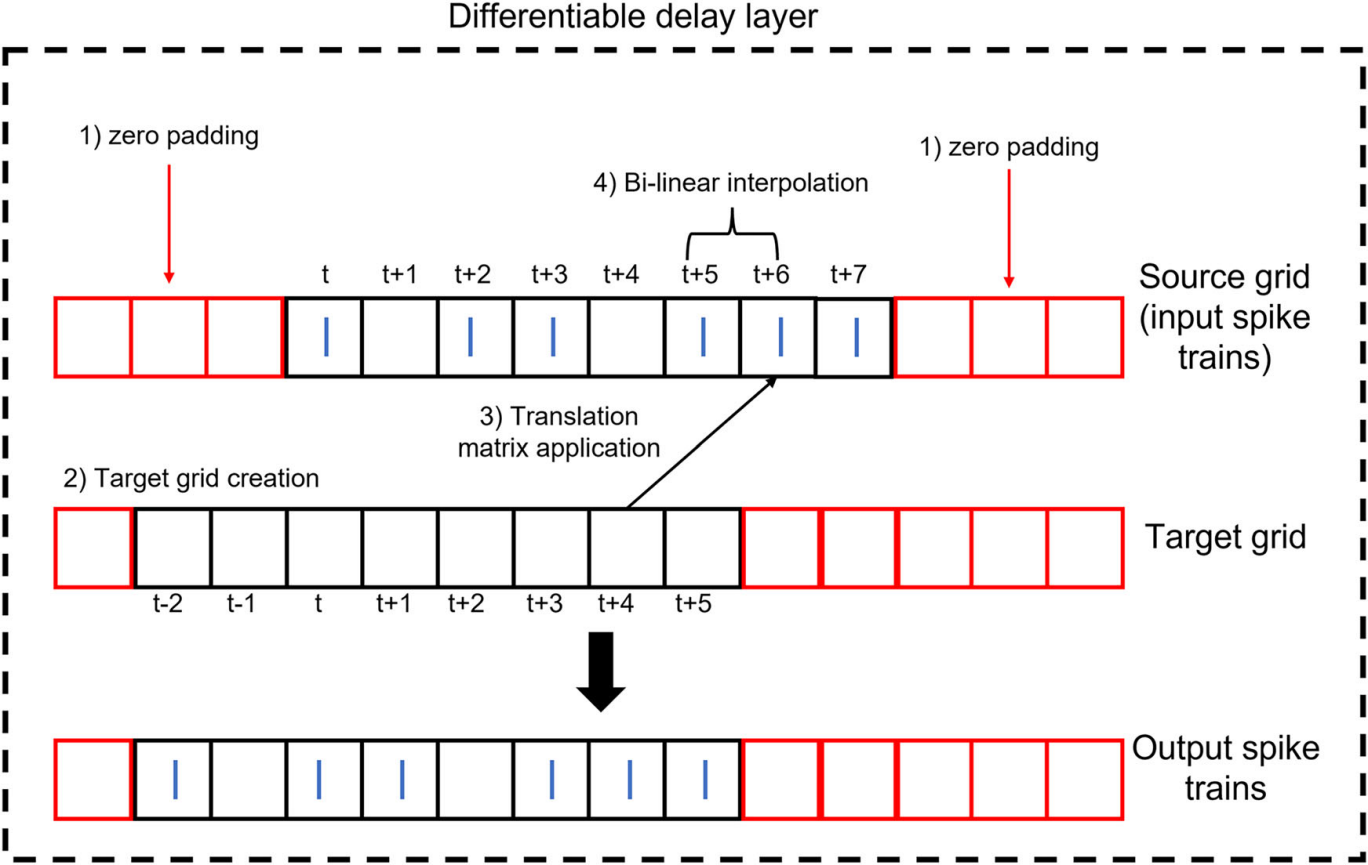
Structure of the DDL. The DDL shifts an input spike train by applying translation then interpolation.

Conventionally, for the spatial transformer, after the projection of the target grid onto the source grid comes the process of interpolation as the transformed pixel might not coincide with a representative one in the source grid/image. Hence, interpolation is performed to estimate the value of the transformed pixel from the surrounding ones. However, applying this process to spike trains can lead to a distortion of the spikes as the allowed values are only 1s and 0s. To avoid this, the translation element *t*_*x*_ should be multiples of the minimum delay. Thus, any transformed spike location from the target grid will find a matching spike location in the source grid. Interpolation (for example, bi-linear) will pick the coincident source spike with a weighting of one and provide zero weighting for any other nearby spikes. This process is summarized visually in Figure 11. The input spike trains to the DDL should be padded by zeros so as to not lose information after translation.

**Figure 12.**
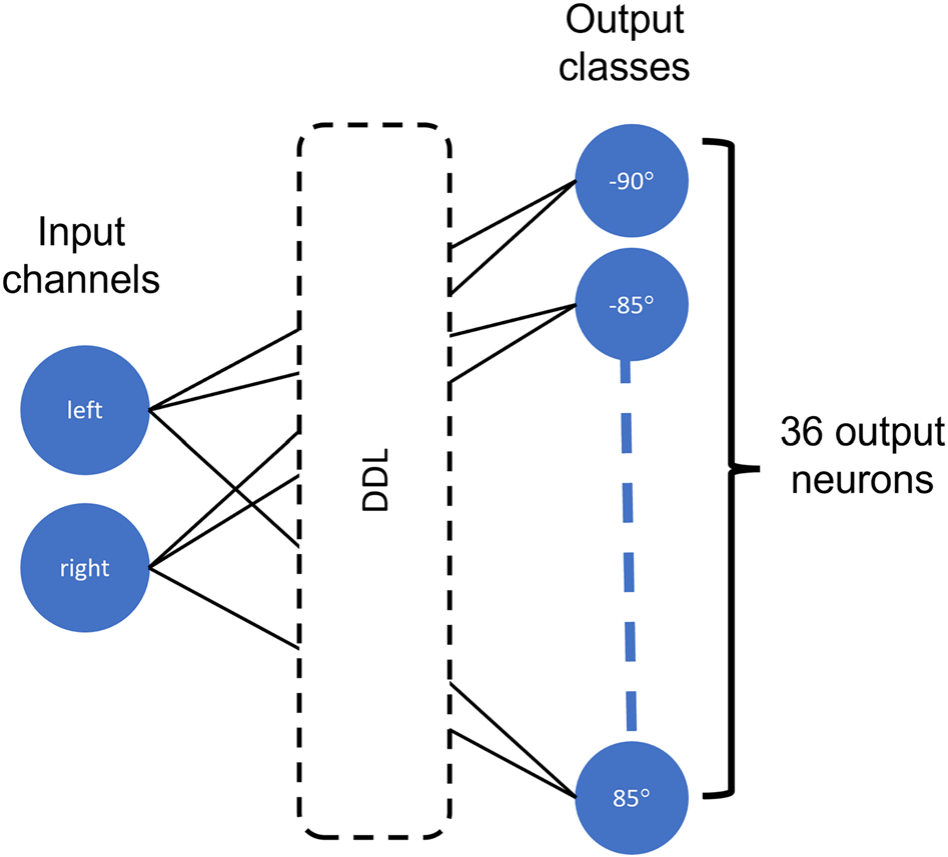
DDL model architecture. The DDL is inserted between the input and output nodes. The output nodes are IPD classes spanning the range [ − 90°, + 85°] in 5° steps.

We will see below that only the DDL is needed to solve the sound localization problem, where the output classes are the target IPD. This network architecture is shown in Figure 12, with the DDL inserted between the input and output nodes. In this case, the weights are set to one and the biases to zero.

The DDL shifts the spike trains in a differentiable way, which modifies the output membrane potential via a multiplicative synapse, described below. Also, to facilitate learning, a non-spiking output was utilized (i.e., the output membrane potential is used and the output neuron does not fire spikes, as in “Methods”).

$$\begin{array}{rl} v_{i} & =-\sum_{t=0}^{T}(u_{1i}(t)-u_{2i}(t){)}^{2}\\ \tau_{m}\frac{du_{xi}}{dt} & =-u_{xi}+S_{x}(t)\\ S_{x}(t) & =\sum_{z=1}^{n}\delta (t-t_{x}^{z}) \end{array}$$

Here, *v*_*i*_ is the voltage at output node *i*, *u*_*xi*_ is the dendritic potential from input *x* ∈ {1, 2} and output node *i*, *τ*_*m*_ is the time constant of a dendritic branch, and *S*_*x*_(*t*) is the spike train of input *x*. The form (*u*_1*i*_(*t*) − *u*_2*i*_(*t*))^2^ looks like a squared error and might, at first glance, seem biologically implausible, but by expanding it as follows we see it is a form of multi-synaptic multiplicative-additive interaction:

$$(u_{1i}(t)-u_{2i}(t){)}^{2}=u_{1i}{\left(t\right)}^{2}-2u_{1i}(t)u_{2i}(t)+u_{2i}{\left(t\right)}^{2}$$

In addition to the DDL, we also use the DCLS (Hammouamri et al., 2024) algorithm, which operates by delaying spike trains through a 1D convolution featuring a single non-zero element, equivalent to the synaptic weight, positioned at the appropriate delay value. This method also uses interpolation to identify the optimal delay, facilitating the learning of delays with weights through backpropagation through time in arbitrarily deep SNNs (see Hammouamri et al., 2024 for details).

#### Results and discussion

**Figure 13.**
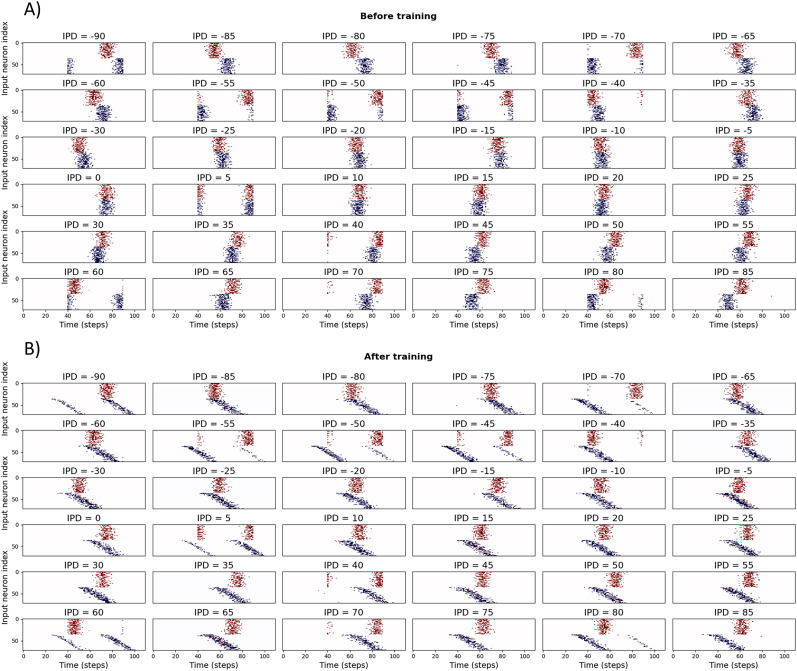
Spike histograms for IPDs ***A***, before and ***B***, after training. Each panel shows the delayed input spikes for a sample sound at a particular IPD. Note that the first half of the spikes (colored in red) have fixed delays, while the second half (blue) are learnable. The shift from a fixed delay to a set of delays can be seen in the transition from vertical to diagonal blue bands of spikes after training.

For the DDL, we discretised IPDs into 36 classes from −90° to +85° in 5° increments. To simplify learning, we fix delays from one ear and only allow delays from the other ear to be learned. This can be seen in switch from the vertical bands of blue spikes in Figure 13*A* before learning to the diagonal bands in Figure 13*B* after learning.

**Figure 14.**
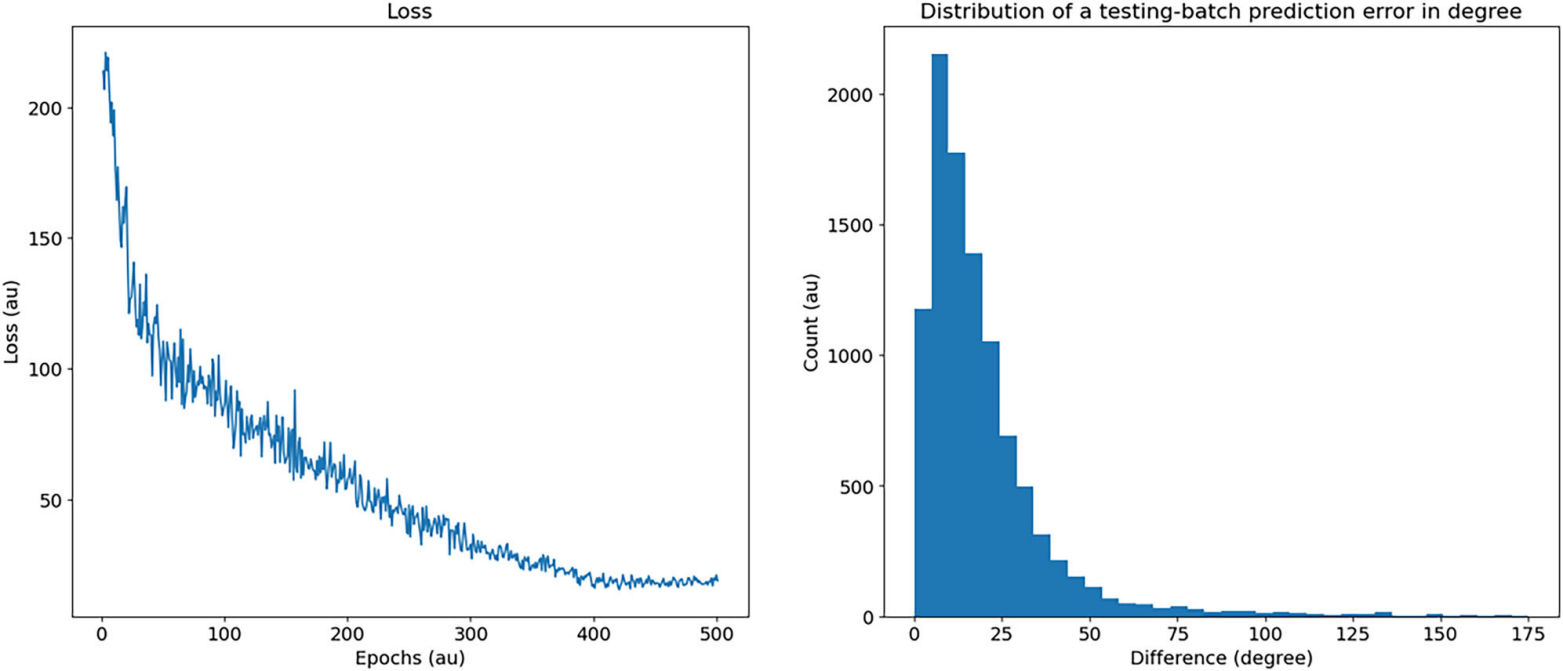
Left: evolution of training loss of the differential delay layer model as a function of the number of training epochs. Right: histogram of absolute errors (in °) after training.

**Figure 15.**
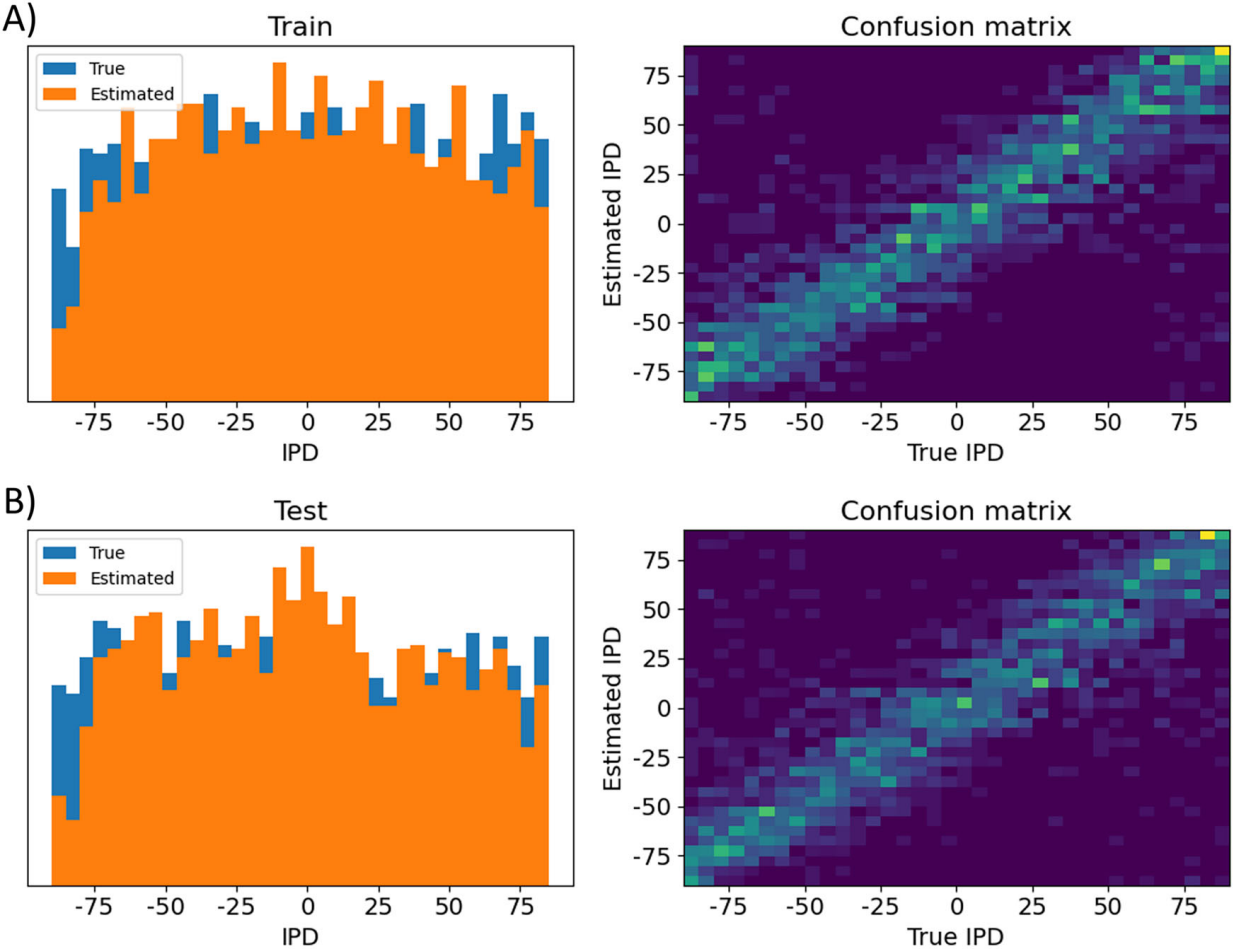
Analysis of classifications by the trained differential delay layer model. Data are shown for errors made on ***A***, the training data set and ***B***, the test data set. Left shows a histogram of the true IPDs (blue) and estimated IPDs (orange). Right shows the confusion matrices on a blue–yellow color scale (so perfect prediction would correspond to a blue image with a yellow diagonal).

Loss curves and the histogram of errors after training are shown in Figure 14. Overall errors correspond to a classification accuracy of 11.8% (compared to a chance level of 2.8%) or a mean absolute error of 19.1°. Confusion matrices are shown in Figure 15.

**Figure 16.**
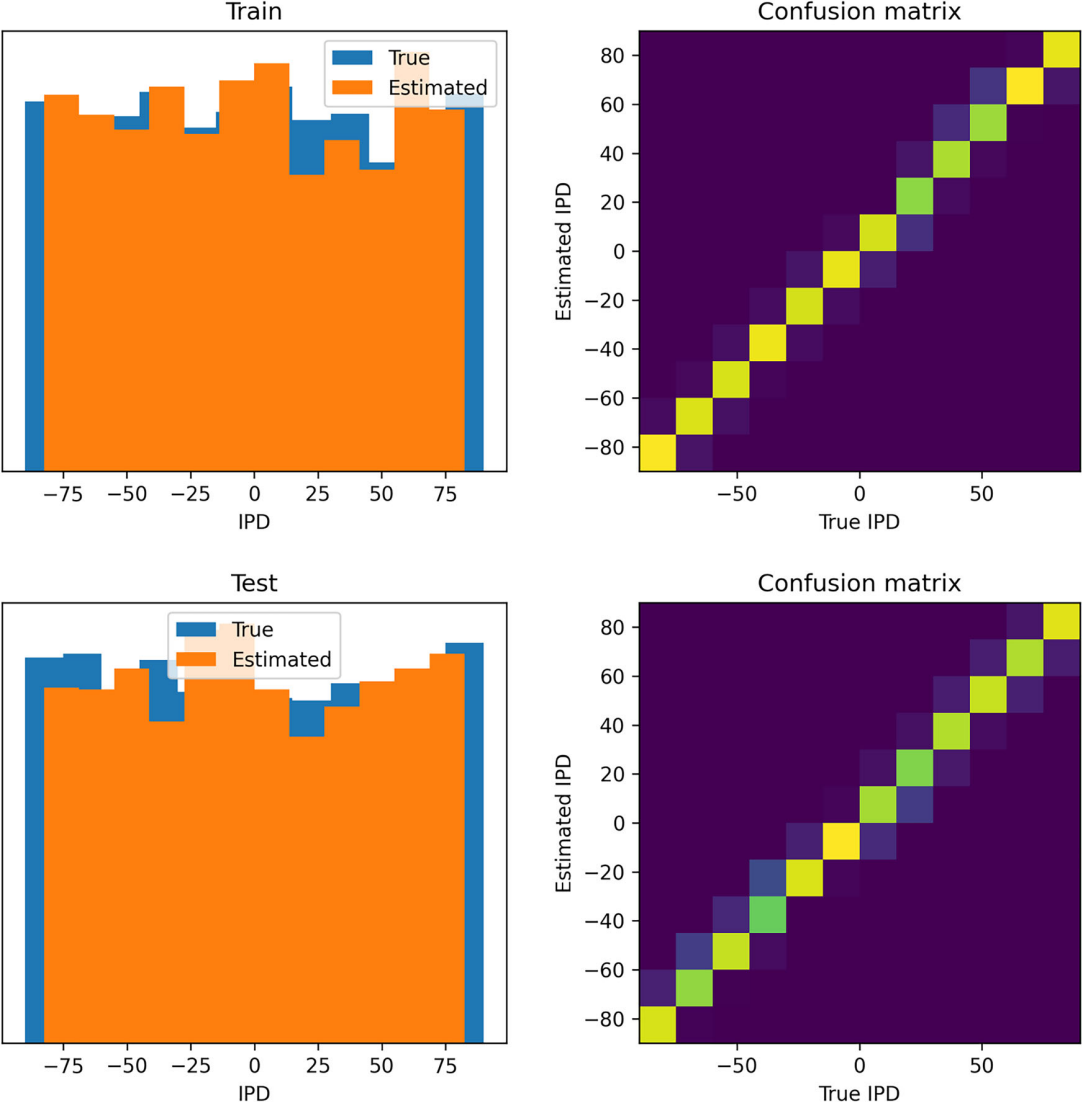
Analysis of classifications by the trained DCLS (DCLS) model. Data are shown for errors made on ***A***, the training data set and ***B***, the test data set. Left shows a histogram of the true IPDs (blue) and estimated IPDs (orange). Right shows the confusion matrices on a blue–yellow color scale (so perfect prediction would correspond to a blue image with a yellow diagonal).

Next we show results for the DCLS (DCLS) algorithm, in this case using 12 IPD classes instead of 36, in Figure 16. Performance of this algorithm for this task was better, with a mean absolute error on the test dataset of 4.2°.

**Figure 17.**
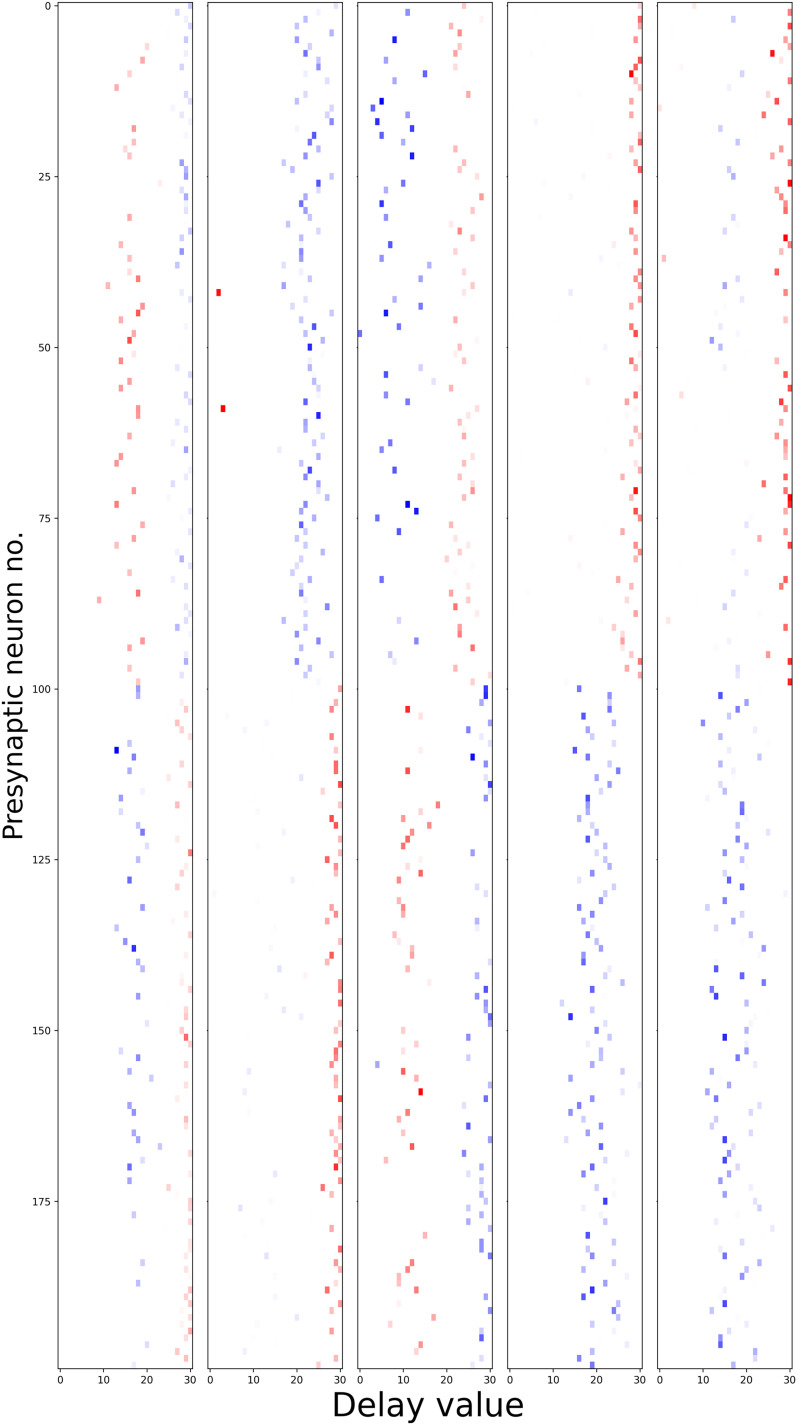
Receptive fields of five randomly chosen neurons in the hidden layer of the DCLS model. The *x*-axis represents the presynaptic neuron index, while the *y*-axis displays the learned delay value. Colors indicate the sign of the weight (blue = excitation, red = inhibition), with transparency denoting magnitude. Excitation and inhibition appear to be spatiotemporally separated.

Learning synaptic delays with weights enables the visualization of the “receptive field” of postsynaptic neurons, as illustrated in Figure 17. Five randomly chosen neurons from the hidden layer are plotted, revealing clear spatiotemporal separation of excitation and inhibition.

### Contralateral glycinergic inhibition as a key factor in creating ITD sensitivity

**Table:** 

Section authors	Francesco De Santis and Alberto Antonietti
Notebooks	Inhibition Model Notebook

#### Highlights

Here, we use a more biologically inspired model to overcome some limitations that have been highlighted in the classic Jeffress model, whose existence, in mammals, is still debated. We focused on the inhibitory inputs to the MSO, which were found both anatomically (there are inhibitory ipsilateral and contralateral pathways) and computationally (in our model) to have a central role for coding ITDs. Experiments with inhibition blocked (by a glycine antagonist: strychnine) show indeed a loss of ITD-coding in the MSO.

#### Introduction

Our starting point was that it is not possible to fully understand ITD sensitivity in the MSO without an understanding of ILD representation in the lateral superior olive (LSO). From an evolutionary point of view, specific to the mammalian phylogenetic tree, the MSO can in fact be considered a refined version of the older LSO (Grothe and Pecka, 2014).

LSO principal cells have a bipolar dendritic tree that receives excitatory input from the ipsilateral ear and inhibitory input from the contralateral one. All mammals appear to apply one common neural strategy for processing ILDs, which consists of subtraction between these two inputs. For instance, neurons in the right LSO will be more excited when sound arrives from the right and more inhibited when sound arrives from the left. The function that describes LSO neurons’ firing rate according to different azimuth angles is usually a sigmoid, presenting the highest values for sound coming from the hemispace ipsilateral to the nucleus, with a steep slope centered on frontal angles (i.e., close to 0°), possibly suggesting a rate-coding strategy for identifying different ILD values with high sensitivity for frontal angles, as suggested by psychoacoustic evidence.

Despite the on-off nature of this subtraction strategy, for which exquisite timing of inhibitory influences appears not to be a key prerequisite, the two major inputs to the LSO are specialized for high-fidelity temporal transmission. An explanation is found in the fact that the subtraction process happening in LSO principal cells is realized in a phase-locked way with respect to the stimulus. This suggests a purely suppressive coincidence mechanism happening at each period of the phase-locked inputs to the LSO (spiking occurs unless there is a binaural coincidence).

The MSO, on the other hand, receives two additional inputs compared to the LSO: a contralateral excitation and an ipsilateral inhibition. As a result of experimental observations, it has been hypothesized that the combination of these four inputs has converted the suppressive coincidence mechanism present in the LSO into an excitatory coincidence mechanism for the detection of ITDs in the MSO (spiking occurs only if binaural coincidence is happening) (Grothe and Pecka, 2014).

We evaluated the activity of these four nuclei (left and right LSO and MSO), using the firing rates of model LSOs as a validation platform for our network, while exploring the impact of certain parameters in the processing of ITDs in the in silico MSO. In order to define a firing rate activity target during this second phase of the work, we used the results from the following two experimental studies:

- Brand et al. (2002) analyzed in vivo recordings from the MSO of the *Mongolian gerbil* and showed how all the 20 neurons tested responded maximally to sounds leading in time at the contralateral ear. Peaks in the firing rate of these neurons were also found for (artificial) ITD values higher than the highest possible ones generated by the gerbil head, which correspond to almost 120 *μ*s for a sound coming at 90° from the contralateral hemispace.
- Pecka et al. (2008) explored how the physical mechanisms underlie the shift in peak activity toward contralateral ITD values. The results showed how the two inhibitory inputs to the MSO, which are not considered in the Jeffress model, play a central role in this process. By blocking the glycinergic inhibition to the MSO neurons by its antagonist, strychnine, the authors observed the loss of the peak shift in all their response activity. With inhibition blocked, all the neuron responses peaked at null ITD values (i.e., 0° azimuth angle). Inhibition was thus shown to have a central role in identifying the ITD values in the MSO. Consequently, in our model, we tried to block these inhibitory inputs to explore the effect that they have on the MSO activity.

Finally, we wanted to understand how the firing rate activity of different neurons in the MSO could be differentiated (i.e., different peak positions in the contralateral space) in order to code for different ITD values and consequently different sound source azimuth angles.

Some additional experimental studies reported how inhibitory inputs, especially the contralateral one, can arrive slightly in advance of the excitatory inputs from the corresponding sides, thus influencing the way ipsilateral and contralateral inputs add up in the MSO neurons and thereby generating a range of maximum responses for contralateral ITD values (Roberts et al., 2013; Myoga et al., 2014). Nevertheless, other work has characterized the hypothesis of anticipation of inhibitory inputs as implausible (vanderHeijden et al., 2013).

In the absence of a clear and shared hypothesis in the literature, we decided to explore further, asking whether or not MSO responses could depend on different shapes of PSPs, both excitatory and inhibitory.

#### Methods

**Figure 18.**
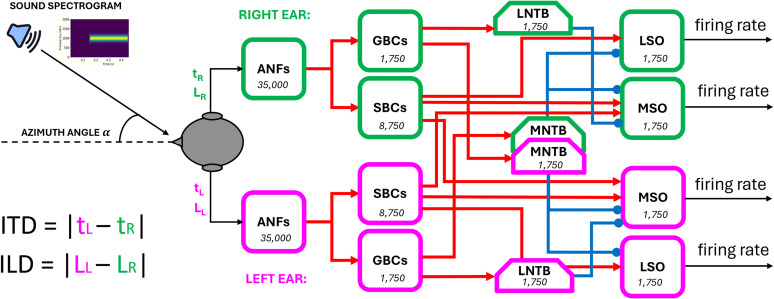
End-to-end brainstem model. Incoming sounds are converted into spectrograms and fed to each ear with a delay *t*_*L*/*R*_ and gain *L*_*L*/*R*_ (modeled as maximum firing rate or count). ANF responses are modeled as phase-locked Gaussian pulse packets. ANF spikes are fed into a network of LIF neurons with the architecture shown, before ultimately being converted into firing rates at the LSO/MSO outputs. The number of neurons for each population is written in the respective block.

We implemented a complex SNN in Python using the NEST Simulator framework (Spreizer et al., 2022). The different neuronal populations composing the brainstem circuit and their interconnections are shown in Figure 18.

The principal inputs to the network are the spectrogram of a sound stimulus arriving at both ears and the azimuth angle from which values of ITD and ILD are computed.

For simplicity, the ITD value is modeled by two ears separated by a fixed width with no head (so the time difference is $w_{\mathrm{head}}\sin\;(\mathrm{Azimuth})/c$ for *c* the speed of sound in air). The ILD value is modeled as a difference in the number of spikes between the left and right auditory nerve fibers (ANFs). This strategy does not take into account the frequency- and distance-specific variability of the ITD and ILD values, however for this study it was sufficient as we only used a single fixed frequency and distance.

The spectrogram covers 20–20 kHz (the whole human audible range) in 3,500 channels, the estimated number of inner hair cells present in the human cochlea. The width of each frequency channel was not constant throughout the range but grew exponentially as in Fettiplace (2023). Spectra were modeled directly as a Gaussian covering 21 channels around the tone frequency, with a constant value over time.

ANFs were modeled as spike trains with Gaussian phase-locked (Yin et al., 2019) spike packets using the pulse_packet_generator built-in NEST “device.” This allowed us to define the number of spikes in each packet (varying accordingly to the ILD), with spike times that are normally distributed with respect to the central time of the pulse. A source of noise was introduced by setting the standard deviation of the random displacement from the center of the pulse equal to 0.1 ms.

All other cell populations except the MSO (described below) were implemented through iaf_cond_alpha models (a simple implementation of a spiking neuron in NEST using integrate-and-fire dynamics with conductance-based synapses and a postsynaptic change of conductance modeled by an alpha function). With this model, we implemented the bushy cells (SBCs and GBCs) located in the anteroventral part of the cochlear nuclei, the glycinergic neurons located in the medial and lateral trapezoidal bodies (MNTB and LNTB), and finally the main cells of the lateral superior olives (LSOs). All the default parameters of this NEST model were kept unchanged (Table 2) apart from the membrane capacitance “C_m,” which was lowered to 1 pF to ensure sufficiently quick membrane time constants as seen experimentally in these neurons Cao et al. (2007).

**Table 2.** Key parameters for the iaf_cond_alpha neural model, including membrane properties, spike-related parameters, and synaptic properties

Parameter	Value	Description
C_m (pF)	250 pF	Membrane capacitance
g_L (nS)	16.6667 nS	Leak conductance
E_L (mV)	−70 mV	Leak reversal potential (resting potential)
refr_T (ms)	2 ms	Duration of the refractory period
V_th (mV)	−55 mV	Spike threshold potential
V_reset (mV)	−60 mV	Reset potential
E_exc (mV)	0 mV	Excitatory reversal potential
E_inh (mV)	−85 mV	Inhibitory reversal potential
tau_syn,exc (ms)	0.2 ms	Synaptic time constant of excitatory synapse
tau_syn,inh (ms)	2 ms	Synaptic time constant of inhibitory synapse

The MSO principal cells were instead implemented through the iaf_cond_beta model. The use of a beta function to replicate the postsynaptic change of conductance allowed us to specify independently the time constants of the rise and fall of the conductance change and thus modify both the excitatory and inhibitory PSP shapes in the MSO. In this way, we could explore different sets of values and attempt to validate our hypothesis about how inhibitory inputs can code for different ITD values in MSO cells, see “Introduction.”

For the validation of the complete brainstem network, including both LSO and MSO of both sides, sound stimuli with frequencies of 100 Hz and 1 s duration from different spatial positions were tested (azimuth angles ranging from −90° to +90° with an interval of 15°). The MSO response was tested both in physiological conditions and with blocked inhibitory inputs, as in the experiments of Brand et al. (2002) and Pecka et al. (2008).

#### Results

**Figure 19.**
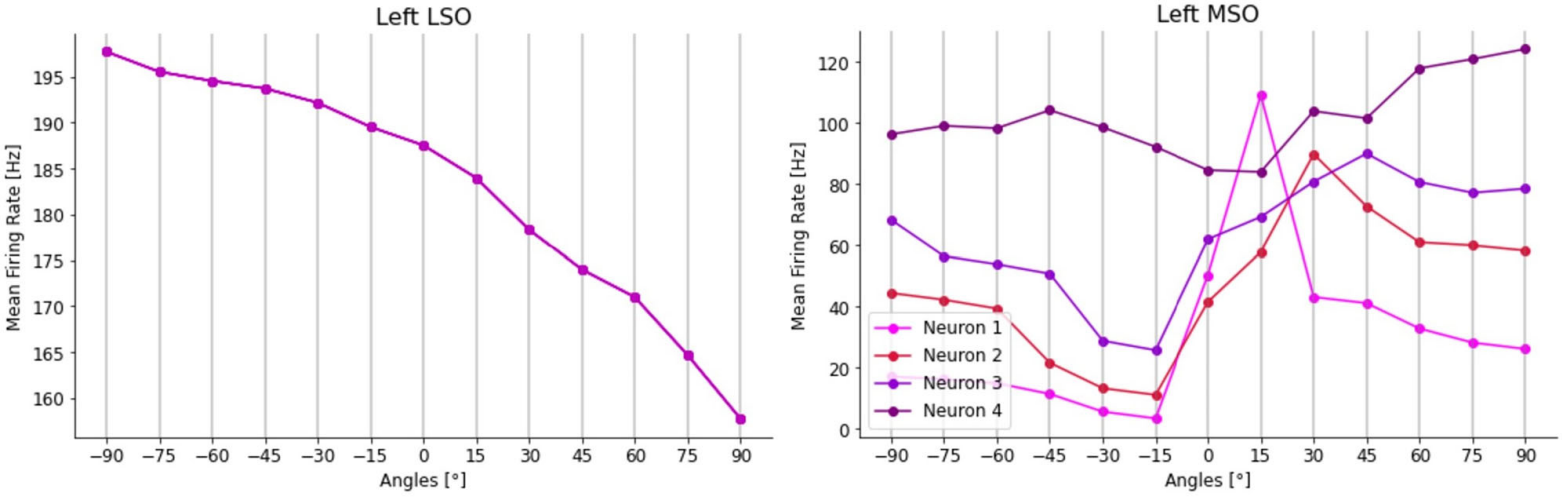
Mean firing rate responses of the left LSO (on the left) and the left MSO (on the right) after stimulation with a 100 Hz pure tone sound for 1 s at different azimuth angles. For the MSO, four neurons with different time constants are shown. Neuron 1 responds maximally for input at +15°, neuron 2 at +30°, neuron 3 at +45°, and neuron 4 at +90°.

Model LSO responses are shown in Figure 19, matching the desired subtraction process described in “Introduction.” Considering for the sake of simplicity the left LSO, when the sound arrives from a source placed at 90° (i.e., right), the right ear receives sounds earlier and more intensely than the left. As the azimuth angle proceeds toward 0° (frontal position), the firing rate of the left LSO increases while maintaining a constant slope. Once past 0°, the firing rate ceases to increase steadily, and the response flattens out to high-rate values. Here, the ipsilateral (left) excitation dominates due to louder sounds (Table 3).

**Table 3.** Brainstem network data about different populations cell types, NEST models used, convergence, number of units, and mean number of cells within a single frequency channel

Cell type	Model	Convergence	Number	Number per frequency channel
ANFs	Poisson generator (devices)	4:1 to SBCs, 20:1 to GBCs	35,000	10
				
SBCs	iaf_cond_alpha	5:1 to LSO PCs, 5:1 to MSO PCs	8,750	2.5
				
GBCs	iaf_cond_alpha	1:1 to MNTB PCs	1,750	0.5
MNTB PCs	iaf_cond_alpha	1:1 to LSO PCs, 1:1 to MSO PCs	1,750	0.5
				
LSO PCs	iaf_cond_alpha	–	1,750	0.5
MSO PCs	iaf_cond_beta	–	7,000	3.5

Model MSO responses are also shown in Figure 19, where the different curves represent the activity of four neurons in the left MSO for which we varied only the value of the time constants of the decay in conductance generated by input spikes for both excitatory (tau_decay_exc) and inhibitory (tau_decay_inh) inputs. We observed that different combinations of these two values provided coding for a specific angle achieved by a peak at different angles (and thus different ITD values) in that cell’s activity. As observed experimentally, all peaks were present in the contralateral sound space, thus confirming the hypothesis that, in contrast to the coding strategy applied in the LSO, higher activity is present in the MSO for sounds from the contralateral space.

**Figure 20.**
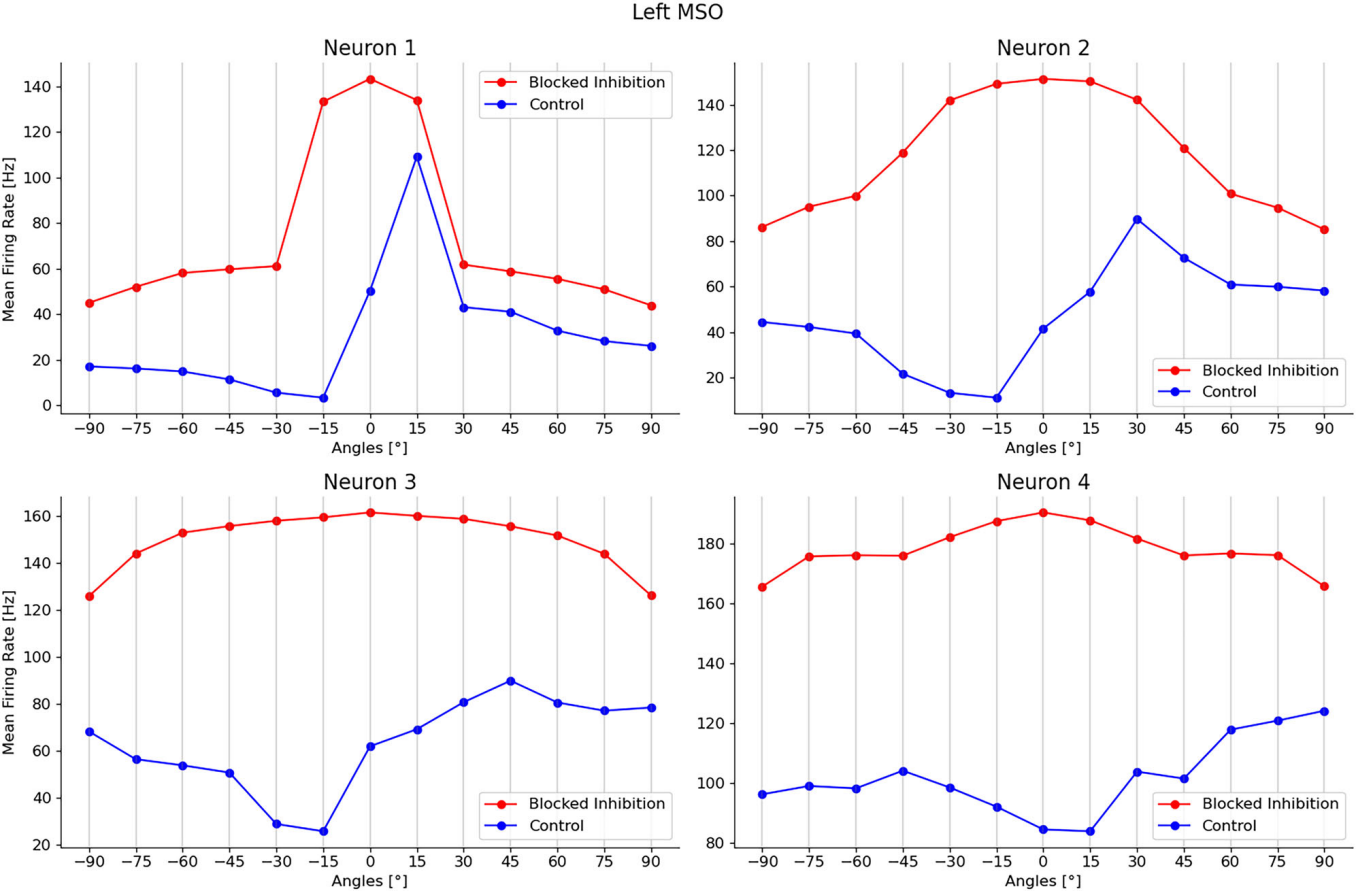
Loss of contralateral ITD peak-coding for the four different neurons in the left MSO: the control condition (with physiological inhibitory inputs) is shown in blue, whereas the curves in red depict the condition with blocked inhibition to the MSO. In the latter scenario, firing rate values are higher with respect to the former and peaks are shifted to null ITD values, so that the coding of each neuron for a specific azimuth angle is now lost.

We simulated MSO cells receiving only excitatory inputs (Fig. 20). A loss in the coding of different contralateral angles is evident from a symmetric firing rate curve, with all the peak values being higher and shifted toward 0° angles with respect to the physiological activity, as measured experimentally in Pecka et al. (2008).

#### Discussion

The computational model developed validates recent theories concerning the processing of the ITDs in the MSO. The peak-coding strategy applied for the identification of contralateral angles in each MSO can be considered a refinement of the rate-based localization of sounds happening in the LSO. As described in “Introduction,” this type of redundancy is also justified by the evolutionary history of spatial hearing mechanisms in mammals. The differentiation in the activity of MSO neurons due to the different time constants of the decay in conductance generated by input spikes was found to be a valid hypothesis for implementing the coding of ITDs. In the end, this work leads to the possible conclusion that strategies similar to those used for the processing of ILDs could also been readapted for the processing of ITDs and that, in the brainstem of modern mammals, these two processes could occur in parallel, merging at a higher level, and thus providing a more refined and complete spatial map.

## Synthesis

Reviewing Editor: David Schoppik, New York University Grossman School of Medicine Decisions are customarily a result of the Reviewing Editor and the peer reviewers coming together and discussing their recommendations until a consensus is reached. When revisions are invited, a fact-based synthesis statement explaining their decision and outlining what is needed to prepare a revision will be listed below. The following reviewer(s) agreed to reveal their identity: NONE.

Given the limited scientific scope of the modeling, we think the title overstates what was done. We suggest revising it to specifically refer to interaural time differences, rather than sound localization more generally:

"Spiking neural network models of interaural time difference extraction via a massively collaborative process.";
